# Supplementary material for: How can primary care enhance end-of-life care for liver disease? Qualitative study of general practitioners’ perceptions and experiences
Source: BMJ Open. 2017 Sep 1;7(8):e017106. doi: 10.1136/bmjopen-2017-017106 (PMC5588936; doi:10.1136/bmjopen-2017-017106)
Supplement: Author's manuscript [file bmjopen-2017-017106.draft_revisions.pdf]

BMJ Open is committed to open peer review. As part of this commitment we make the peer review history of every article we publish publicly available.

When an article is published we post the peer reviewers' comments and the authors' responses online. We also post the versions of the paper that were used during peer review. These are the versions that the peer review comments apply to.

The versions of the paper that follow are the versions that were submitted during the peer review process. They are not the versions of record or the final published versions. They should not be cited or distributed as the published version of this manuscript.

BMJ Open is an open access journal and the full, final, typeset and author-corrected version of record of the manuscript is available on our site with no access controls, subscription charges or pay-per-view fees (http://bmjopen.bmj.com).

If you have any questions on BMJ Open's open peer review process please email info.bmjopen@bmj.com

BMJ Open

How can primary care enhance end-of-life care for liver disease? A qualitative study of general practitioners' perceptions and experiences

Journal:	*BMJ Open*
Manuscript ID	bmjopen-2017-017106
Article Type:	Research
Date Submitted by the Author:	31-Mar-2017
Complete List of Authors:	Standing, Holly; Newcastle University, Institute of Health & Society<br>Jarvis, Helen; Newcastle University, Institute of Health & Society<br>Orr, James; Newcastle University, Institute of Cellular Medicine<br>Exley, Catherine; Northumbria University, Faculty of Health and Life Sciences<br>Husdon, Mark; Freeman Hospital; Newcastle University, Institute of Cellular Medicine<br>Kaner, Eileen; Newcastle University, Institute of Health and Society<br>Hanratty, Barbara; Newcastle University, Institute of Health and Society / Newcastle University Institute for Ageing
**Primary Subject Heading**:	General practice / Family practice
Secondary Subject Heading:	Gastroenterology and hepatology, Palliative care
Keywords:	PALLIATIVE CARE, liver diseases, terminal care, patient care

SCHOLARONE™ Manuscripts

For peer review only

How can primary care enhance end-of-life care for liver disease? A qualitative study of general practitioners' perceptions and experiences

SUBMISSION TO: BMJ Open

AUTHORS
Holly Standing, Helen Jarvis, James Orr,
Catherine Exley, Mark Hudson, Eileen Kaner, Barbara Hanratty

Dr Holly C Standing
Institute of Health and Society
Newcastle University,
Holly.standing@newcastle.ac.uk

Dr Helen Jarvis
Institute of Health and Society, Newcastle University, Biomedical Research Building,
Campus for Ageing and Vitality, Newcastle upon Tyne NE4 5PL
Helen.jarvis2@newcastle.ac.uk

Dr James Orr
Institute of Cellular Medicine, Newcastle University
James.orr@newcastle.ac.uk

Professor Catherine Exley
Faculty of Health and Life Sciences,
Northumbria University,
Newcastle upon Tyne NE1 8ST
Catherine.exley@northumbria.ac.uk

Dr Mark Hudson
Freeman hospital, High Heaton, Newcastle upon Tyne, NE7 7DN
Institute of Cellular Medicine, Newcastle University
Mark.Hudson@nuth.nhs.uk

Professor Eileen Kaner
Institute of Health and Society, Newcastle University, The Baddiley-Clark Building
Richardson Road, Newcastle upon Tyne NE2 4AX
Eileen.kaner@newcastle.ac.uk

Professor Barbara Hanratty*
Institute of Health and Society, Newcastle University, Biomedical Research Building,
Campus for Ageing and Vitality, Newcastle upon Tyne NE4 5PL
Barbara.hanratty@newcastle.ac.uk

***Author for correspondence: Barbara Hanratty**

Word count 3978 (including quotes)
Key words (MeSH) : terminal care, palliative care, liver diseases, patient care

For peer review only

For peer review only - http://bmjopen.bmj.com/site/about/guidelines.xhtml

Abstract

Background: Liver disease is the third commonest cause of premature death in the UK. The symptoms of terminal liver disease are often difficult to treat, but very few patients see a palliative care specialist, and a high proportion die in hospital. Primary care has been identified as a setting where knowledge and awareness of liver disease is poor. Little is known about general practitioners' (GPs) perceptions of their role in managing end-stage liver disease.

Objective: To explore GPs' experiences and perceptions of how primary care can enhance end-of-life care for patients with liver disease.

Design: Qualitative interview study, thematic analysis

Participants: Purposive sample of 25 GPs from five regions of England

Results: GPs expressed a desire to be more closely involved in end-of-life care for patients with liver disease, but identified a number of factors that constrained their ability to contribute. These fell into three main areas; those relating directly to the condition, (symptom management and the need to combine a palliative care approach with ongoing medical interventions); issues arising from patients' social circumstances (stigma, social isolation and the social consequences of liver disease); and deficiencies in the organisation and delivery of services. Collaborative working with support from specialist hospital clinicians was regarded as essential, with GPs acknowledging their lack of experience and expertise in this area.

Conclusions: End-of-life care for patients with liver disease merits attention from both primary and secondary care services. Development of care pathways and equitable access to symptom relief should be a priority.

For peer review only - http://bmjopen.bmj.com/site/about/guidelines.xhtml

Abstract word count 249

Stength and limitations of this study:

- To the best of our knowledge this is the first study to focus on the experiences' of primary care clinicians around managing end-of-life liver disease.
- GPs were recruited from a range of both rural and urban UK locations.
- Use of Semi-structured interviews allowed us to collect detailed descriptions of GPs' experiences of managing end-of-life liver disease.
- The study was conducted by a team of experienced researchers with a range of disciplinary backgrounds including clinicial experience.
- The findings of our study are based on the reported knowdlege and experiences' of self-selecting participants, their views may not be transferable to the wider GP population.

Introduction

End stage liver disease is an important challenge for providers of palliative and end-of-life care. Death rates have increased 400% since 1970, and it is now the third commonest cause of premature death in the UK.[1, 2 3] In 2012, around 11,000 deaths were attributed to liver disease in the UK. Patients have a complex and heavy symptom burden that is often poorly treated [4 5] and the psychosocial needs of patients and families may be considerable.[6] The majority of patients present late to services, when liver disease is irreversible and around 70% die in hospital.[7] The typical clinical course, of gradual decline punctuated by episodic decompensation, may mean that treatment is focused on prolonging life through means such as transplantation, and a palliative care approach is rarely considered.[8] Liver

For peer review only - http://bmjopen.bmj.com/site/about/guidelines.xhtml

transplantation is the only curative treatment in end stage liver disease but many patients will be unsuitable due to co-morbidities, advancing age or ongoing alcohol consumption. Some evidence suggests that patients who are considered and rejected for organ transplantation, are unlikely to receive any palliative care.[9]

There is a growing international consensus that end-of-life care for patients with liver disease requires improvement.[4 10-12] Primary care has been identified as a specific area where care could be enhanced, particularly around the discussion of palliative care needs with patients, the inclusion of patients on palliative care registers, and improving communication between primary and secondary care.[8 13] Primary care professionals are well placed to provide holistic care that patients dying with liver disease need, but are seldom involved. In addition, GP care for patients dying with liver disease is not rated highly by bereaved relatives.[14]

Little is known about how health professionals in primary care see their role in end-stage liver disease, or what they view as the main barriers to providing high quality care. This study intends to contribute to our understanding of this patient group and to inform the development of appropriate services. The aim is to explore GP experiences and perceptions of end-of-life care for patients with liver disease.

Methods

This study employed qualitative methods, involving semi-structured interviews with GPs from five geographical areas within England.

For peer review only - http://bmjopen.bmj.com/site/about/guidelines.xhtml

For peer review only

Participants

Participants were recruited via National Institute for Health Research Clinical Research Networks and local networks of GP practices in the London, Thames Valley, Wessex, Yorkshire and the North East. Email invitations were sent to GP practices within these networks, and GPs who wished to participate then contacted the research team. Following the first phase of interviews, participants were purposively sampled in order to provide a wide range of clinical experience and degree of familiarity with liver disease, this involved targeting individuals in areas of high and low prevalence.

Data collection

A semi-structured interview guide was developed to cover issues identified through a review of the literature; including GPs' experiences of identifying and managing end-of-life liver disease. This was a 'living' document that evolved throughout data collection to allow exploration of emerging areas. Interviews were conducted face-to-face (n=2) or over the telephone (n=23). Interviews lasted between 15 and 50 minutes and were all conducted by the first author between March and August 2016. Field notes were taken to aid subsequent analysis. Informed consent was obtained from all participants. Data collection ceased when no themes were emerging from the interviews (see below for further detail).

For peer review only - http://bmjopen.bmj.com/site/about/guidelines.xhtml

Data analysis

Audio recordings of interviews were transcribed verbatim by an independent transcription company; transcripts were checked for accuracy by listening again to each recording. The NVivo 10 software package was used to manage the data.

A thematic analysis was conducted based on the approach of Glaser and Strauss [15] The analysis was iterative, with data collection and analysis running concurrently throughout the study. Analysis of early transcripts informed the interview schedule for later interviews and each transcript was re-examined in light of subsequent interviews. The researchers began by familiarising themselves with the data through detailed reading of the transcripts followed by line-by-line coding.[16] Field notes taken during data collection were used throughout analysis to enhance the reflective process.

Several quality control measures were employed to ensure trustworthiness of the data. A proportion of the transcripts (20%) were coded independently by three researchers, before coming together to compare their analysis. Data analysis and emerging themes were also discussed amongst the wider research team, which included individuals with clinical expertise in general practice and hepatology.

For peer review only

Findings

Twenty five GPs were interviewed. The majority had been qualified as GPs for five or more years, but few (4/25) had any specialist hepatology or gastroenterology training or experience. Participant characteristics are shown in table 1.

For peer review only - http://bmjopen.bmj.com/site/about/guidelines.xhtml

Table 1 here

Four themes were identified from the data analysis: the role of the GP, acknowledging and accepting end-of-life, collaborative care pathways, and social relationships and consequences. The quotations presented below are illustrative, representing typical participant responses and demonstrating the varied viewpoints.

The role of the GP

In this study, few of the interviewees had extensive first-hand experience of managing patients with liver disease at the end-of-life. Those who did, reported that they managed such cases infrequently, and some years may go by without them seeing a case.

> *"[We manage] a lot of dying people, but not from the hepatology point of view. I don't know if they tend to be managed in hospital predominantly more than in primary care? That's a possibility, I guess."* (GP 7)

Some of the interviewees attributed their lack of expertise and experience of caring for liver patients at the end-of-life to a reluctance amongst hospital clinicians to relinquish control.

> *"There are some conditions, like liver disease, renal failure, they [patients] all just end up dying in hospital for some reason. I don't*

For peer review only - http://bmjopen.bmj.com/site/about/guidelines.xhtml

know whether it's the hospital consultants that don't want to let them go home… They need to let go and make sure there's a palliative care plan in place…they don't do it." (GP 3)

The limited contact between GPs and patients dying with liver disease was attributed to an unpredictable disease trajectory with periods of stability and decompensation, and to patients remaining under the care of hospital services in their last weeks and months. The GPs in this study shared a view that end-of-life care is a core component of primary care, and interviewees questioned how appropriate it was for specialist hospital clinicians to take a lead in palliative care. Patients with liver disease were not regarded as distinct or different from patients dying with other conditions, and a number of GPs expressed a desire for greater involvement in their end-of-life care. Some participants implied that primary care involvement may support more patients to die at home, rather than in hospital.

"I think primary care probably is best placed, in most cases, to look after people- well not only for that [liver disease], for most end-of-life care issues. So, yeah, I think the GP is probably the most important person in the sense that they can bear in mind what the specialists have advised, but at the end of the day, try and keep some of these patients at home rather than having to have them admitted acutely." (GP 14)

Acknowledging and accepting end-of-life

Judging when a patient with liver disease is nearing the end-of-life was perceived to

For peer review only - http://bmjopen.bmj.com/site/about/guidelines.xhtml

be a particular challenge. Communication about prognosis and the age of patients were identified as important factors. Some of the GPs reflected on how management decisions taken in hospital send out messages that influence care provided in the community. Continuing to pursue active treatment may convey optimism about the patient's life expectancy. Specifically, GPs referenced occasions where patients had been placed on the waiting list for a liver transplant, which the patient saw as offering them a second chance at life, even though they were critically unwell and may die whilst waiting for an organ. Patients with a primary liver condition are often younger than the typical palliative care patient. It is likely that clinicians are more reluctant to give up on active treatment for younger patients, whilst patients and families may also struggle to accept that the end-of-life is approaching.

> *"Those patients where it's a, kind of, grey area about whether they're end-of-life or not. And I think that mainly stems from the fact that if it's a young patient, it's more difficult for health care professionals, the patients themselves, and families, to actually accept that the person's dying"* (GP1)

Mixed or uncertain messages may mean that care is compromised, if no one engages the patient in discussions about the end-of-life, and a palliative approach is never considered.

> *"I suppose, looking back it really was palliative care but they [secondary care] put him on the transplant list because he's given up alcohol and there was still this hope. So therefore we didn't really*

For peer review only - http://bmjopen.bmj.com/site/about/guidelines.xhtml

> *realise he was going to die as quickly as he did"* (GP 11)

There was a shared feeling amongst interviewees, that specialists should provide clear messages about patients' prognoses, so that GPs can adopt an appropriate management plan. At present, hospital specialists were perceived as failing to take responsibility for identifying patients as end-of-life, and this had a detrimental impact on primary care.

> *"I feel that it should be made compulsory for the secondary care, tertiary care sectors, when they discharge, or when they're seen in the patient clinic, [to] prognosticate, … then we can initiate also, the discussion with the patient, in a much more positive way."* (GP 12)

Collaborative care pathways

Supporting patients with liver disease was seen as a collaborative effort, with GPs acknowledging their need for specialist guidance, particularly when managing end-of-life complications. Ascites was the most commonly mentioned and problematic symptom experienced by patients with end-stage liver disease, requiring drainage in hospital. Experiences of GPs in this study suggest that ease of access to this procedure was highly variable. In some areas, pathways had been negotiated and patients could be directly admitted to an appropriate ward. In others, GPs described their concern at having to send patients to accident and emergency (A&E) departments. Failure to arrange prompt access to treatment caused distress and was a major source of adverse experiences during end-of-life care.

For peer review only - http://bmjopen.bmj.com/site/about/guidelines.xhtml

> *"We had a nightmare. He was building up litres of ascitic fluid on his tummy every week or week to 10 days, and every time the hospital had to send him acutely, new, to A&E and he had to sit in A&E for hours. I was speaking to the liver specialist ... He needed regular reviews and eventually they agreed to do it two weekly but even that wasn't enough, it was building up and he was ending up going in as an emergency every week."* (GP 11)

Where appropriate care pathways were not in place, interviewees suggested that they were needed, to reassure the patient and GPs that support is available when required.

A number of participants suggested that a specialist nurse may hold the key to more collaborative management of liver patients. They could act as an intermediary between primary and secondary care, negotiating priorities and ensuring effective and easy communication.

> *"It often helps when there is direct access to, say, a nurse specialist in a field, or there is some other point of contact in secondary care that say a family or the patient themselves can call directly for advice."* (GP 14)

Although some GPs had encountered specialist nurses working in this type of role, this was not a common experience. Unfavourable comparisons were made between the services available for patients with liver disease, and other conditions, such as cancer. Participants highlighted the potential benefit to patients and families, of

For peer review only - http://bmjopen.bmj.com/site/about/guidelines.xhtml

having a specialist point of contact in the community, including prompt access to advice and alleviation of fears and concerns.

Social relationships and consequences

GPs in this study argued that people with liver disease had many of the same primary care needs as patients with other life-limiting conditions. However, the severity of symptoms in end-of-life liver disease was felt to be different. Some of the GPs acknowledged the potentially damaging impact on the patient's family, of seeing their relative die at home.

> *"I think there is quite a strong push to keep people at home. Whether that's right or wrong, I don't know really. If they've got ascites or portal hypertension, you know, they've got the risk of vomiting blood and all the rest of it. Or they have been vomiting blood. I'm not massively keen on keeping people at home because it's just a rubbish picture in the mind of everybody, I think, you know, the family left behind."* (GP 17)

Families were perceived to be in need of support themselves, which was an additional role for primary care. GPs described examples of relatives requiring frequent contact and reassurance as the patient's condition deteriorated. The GPs in this study differed in their attitudes towards these demands. Some took a holistic view to the management of palliative patients, believing that these were part of the standard practice of primary care.

For peer review only - http://bmjopen.bmj.com/site/about/guidelines.xhtml

For peer review only

"I think when we talk about palliative care it's not just a single person who's the patient, it's about supporting and managing the family and helping them through that bereavement stage because it starts right at the diagnosis and they have to go through that journey. Death is a part of life and giving them that support." (GP 10)

However, others felt that attending to the needs of patients' families was an extra burden on their already overstretched resources.

Limited social support and unfavourable social circumstances were often mentioned as significant issues for patients with liver disease, particularly when alcohol or drug misuse were factors. Several GPs referred to the 'chaotic' lifestyles of this patient group, and resulting vulnerability to social isolation. Behaviours associated with addiction were perceived to lead to the breakdown of the patients' social networks, leaving few, if any people to provide support or care.

"the demographics of the alcohol dependent ones, who have often, for various reasons and due to the nature of their disease, have become quite isolated, they have not got many people around them and so they don't have that support. They require much more organisation and support in the background, so we make sure that they do have that support." (GP 10)

Without alternative sources of support, socially isolated patients were believed to place extra demands on GPs and other health services. Even when social networks

For peer review only - http://bmjopen.bmj.com/site/about/guidelines.xhtml

were maintained, there could be a dearth of responsible caregivers, as friends and family often shared the problems of addiction and poor health.

> *"I can think of a couple of our households where maybe spouses and partners may have liver cirrhosis themselves. I can think of two couples – well, one person who died two years ago. His wife has chronic liver disease as well."* (GP 4)

One of the most important consequences of social isolation, was that patients had fewer choices over where they spent the end of their life. Without anyone to monitor their condition, they were more likely to be admitted to hospital, and die there.

Liver disease is a potentially stigmatising condition, particularly when the underlying cause is alcohol or substance misuse. Several of the GPs suggested that there is often an assumption within the patient's community that liver disease is self-induced and they were culpable, even when substance misuse or alcohol are not factors.

> *"I think it's a huge problem for people that have liver disease and look like they have liver disease and people assume it's related to alcohol when, in fact, it might be due to auto-immune causes or other forms of cancer or something like that or hepatitis as well."* (GP 23)

For peer review only - http://bmjopen.bmj.com/site/about/guidelines.xhtml

For peer review only

This assumed culpability has implications for the degree of support and sympathy that the patient, and their families, receive. GPs also suggested that stigma could hinder patients' acceptance of their prognosis, which in turn made the management of their condition more challenging. As such, care of liver patients should include psychological and social services.

> *"I think, inevitably and sadly, there is a stigma associated with liver disease, and hence, that's why the psychological support is really important"* (GP 25)

However, some commented that stigmatisation occurred early in the patients' illnesses, and to address this, changes would be needed well before end-of-life care was being considered.

Discussion

This study provides insights into the challenge faced by general practitioners providing end-of-life care for patients with chronic liver disease. Many GPs expressed a desire to be more closely involved, but identified a number of factors that constrained their ability to contribute. These fell into three main areas; those relating directly to the condition, (symptom management and the need to combine a palliative care approach with ongoing medical interventions); issues arising from patients' social circumstances (stigma, social isolation and the social consequences of liver disease); and deficiencies in the organisation and delivery of services. Collaborative working with support from specialist hospital clinicians was regarded as essential, with GPs acknowledging their lack of experience and expertise in this area.

Strengths and limitations

For peer review only

For peer review only - http://bmjopen.bmj.com/site/about/guidelines.xhtml

To our knowledge this is the first study to focus on the experiences of primary care physicians in managing patients with end-stage liver disease. Our interviewees were drawn from rural and urban areas in five different regions in England, and working with a diverse range of communities. The relatively large number of GP participants and varying levels of experience, expertise and interest in the subject, is a particular strength of the study. With our qualitative design, we were not seeking generalisability, but the diversity of the participants increases our confidence that we have not overlooked important issues. The majority of interviews were conducted by telephone, which may explain the ease and speed with which we recruited participants, despite not offering any financial incentives. Use of the telephone is thought to have promoted unguarded responses, but we acknowledge that it can be more difficult to develop rapport in the absence of nonverbal cues and other facets of face to face communication. However, we do not believe that this was a problem, as GPs provided rich and insightful accounts of their experiences.

Comparison with other work

Our findings are consistent with recent research from Scotland that included interviews with eight GPs along with other health care professionals. Communication with secondary care, lack of expertise and limited confidence in prognostication were all identified as concerns.[13] Accurate assessment of prognosis in liver disease is difficult given the unpredictable disease course. In some aspects this is similar to other diseases characterised by episodes of decompensation, such as heart failure. However, liver disease presents the additional challenge that recompensation and improved liver function may be achieved in certain patients, such as those who achieve abstinence from alcohol. A recent review of palliative care guidelines in

For peer review only - http://bmjopen.bmj.com/site/about/guidelines.xhtml

heart failure and chronic obstructive pulmonary disease described wide variation in how patients are identified for palliative care, and attributed this, in part, to the unpredictable disease course and the consequences for care planning.[17] In common with liver disease, acknowledgement and development of end-of-life care has been relatively recent for these conditions.[17]

The GPs in our study agreed that, at the end-of-life, patients with liver disease ideally need primary care and hospital specialists to work closely together. GPs are more likely to have an established relationship with the patient and a greater understanding of their social situation and needs, whereas specialists offer expert knowledge on liver disease and treatment options. They highlighted the importance that primary care physicians place on being able to provide a coordinating role, but only when supported by members of the specialist teams. Managing complex and unusual symptoms, or judging when to introduce a palliative care approach, for example, all benefit from collaboration. The advantages of a multidisciplinary approach have already been well documented in the palliative care literature.[18 19] Several recent reviews on end-stage liver disease have also advocated this approach.[4 5 11]

This study highlights the complexity of caring for patients with end-stage liver disease. Expertise in acute medicine and palliative care are essential, but patients and families also need sensitive and practical responses to their psycho-emotional and social concerns, including stigma related to the perceived self-inflictedness of the disease, social isolation and lack of income. Such generalist expertise and a holistic, person-centred approach are the foundations of primary care. Community-

For peer review only

For peer review only - http://bmjopen.bmj.com/site/about/guidelines.xhtml

based services already play an important role at the end-of-life for patients with many different, complex conditions. However, this seldom includes people dying with liver disease. Greater involvement of community services would be expected to enhance the quality and appropriateness of palliative and terminal care for these patients. As the number of deaths from chronic liver disease increases, it may be increasingly necessary, in order to limit the burden on hospital teams.

Some of the barriers to GP care for patients with end-stage liver disease may be addressed by collaboration and communication between liver specialists and primary care. Prompt and equitable access to treatment could be enhanced in a number of ways. Specific innovations, such as the development of clear patient pathways, specialist heptology nurses in the community or district nurses trained to deal with liver disease complications, all require resources. Specialist treatments such as paracentesis could be delivered in locations such as community hospitals or hospices, where they are available, to reduce disruption to patients' lives.

Conclusion

Our study suggests that end-of-life care for patients with liver disease requires attention. Liver disease appears to pose management challenges in end-of-life care with a combination of complicated social situations and symptoms. Services tailored for these patients should build on the similarities with other conditions, but also reflect the differences. The adverse social consequences of illness for these patients and their families, may be particularly significant. Further research is needed to fully understand the burden on families and services. As health services seek greater integration with social care, improving care for patients with end-stage liver disease should be a priority.

For peer review only

Acknowledgements

We are grateful to the participants who generously gave their time to this study.

Ethics Approval

This study involved no patients and was assessed by the Health Research Authority and Newcastle University Research Ethics (Ref 188275).

Authors contribution

BH, EK, MH, JO, and CE designed the study. HS carried out the interviews. HS undertook the main analysis supported by BH and HJ. HS, HJ and BH drafted the manuscript, and all authors commented and approved the final version.

Funding statement

This work was supported by the NIHR School for Primary Care grant number 262.

Competing interests

We have read and understood BMJ policy on declaration of interests and declare that we have no competing interests.

Data sharing agreement

We do not have any additional unpublished data that can be shared.

References

1. World Health Organisation. WHO European health for all database. 2012. http://data.euro.who.int/ (accessed 22 January 2017).
2. Office National Statistics. Deaths registered in England and Wales: 2015. 2016; (accessed 9th September 2016). https://www.ons.gov.uk/peoplepopulationandcommunity/birthsdeathsandmarriages/deaths/bulletins/deathsregistrationsummarytables/2015 (accessed 22 January 2017).
3. University of Washington. The Liver Imaging Atlas: An Online Reference for Liver Imaging. 2017; 2017(22 January 2017). liveratlas.org (accessed 22 January 2017).
4. Boyd K, Kimbell B, Murray S, et al. Living and dying well with end-stage liver disease: Time for palliative care? Hepatology 2012;**55**(6):1650-51.
5. Roth K, Lynn J, Zhong Z, et al. Dying with end stage liver disease with cirrhosis: insights from SUPPORT. Study to Understand Prognoses and Preferences for Outcomes and Risks of Treatment. J Am Geriatr Soc 2000;**48**(5 Suppl):S122-30.

For peer review only - http://bmjopen.bmj.com/site/about/guidelines.xhtml

6. Kimbell B, Murray SA. What is the patient experience in advanced liver disease? A scoping review of the literature. BMJ Supportive & Palliative Care 2013;**bmjspcare-2012**.
7. National End of Life Care Intelligence Network. Deaths from Liver Disease: Implications for end of life care in England. 2012. http://www.endoflifecare-intelligence.org.uk/resources/publications/deaths_from_liver_disease (accessed 27 Feburary 2017).
8. Kendrick E. Getting it Right Improving End of Life Care for People Living with Liver Disease. London: National End of Life Care Programme, 2013.
9. Poonja Z, Brisebois A, van Zanten SV, et al. Patients with cirrhosis and denied liver transplants rarely receive adequate palliative care or appropriate management. Clin Gastroenterol Hepatol 2014;**12**(4):692-98.
10. Larson AM. Palliative Care for Patients with End-Stage Liver Disease. Curr Gastroenterol Rep 2015;**17**(5):18.
11. Walling AM, Wenger NS. Palliative care and end-stage liver disease. Clin Gastroenterol Hepatol 2014;**12**(4):699-700.
12. Potosek J, Curry M, Buss M, et al. Integration of Palliative Care in End-Stage Liver Disease and Liver Transplantation. Journal of Palliative Medicine 2014;**17**(11):1271-77.
13. Kimbell B, Boyd K, Kendall M, et al. Managing uncertainty in advanced liver disease: a qualitative, multiperspective, serial interview study. BMJ open 2015;**5**(11):e009241.
14. Office of National Statistics. National Survey of Bereaved People (VOICES). 2015. (accessed 27 Feburary 2016).
15. Glaser BG, Strauss A. *The Discovery of Grounded Theory: Strategies for Qualitative Research*. Chicago: Aldine Publishing Co, 1967.
16. Van Manen M. Practicing phenomenological writing. Phenomenology and Pedagogy 1984;**2**(1):1-34.
17. Siouta N, Van Beek K, van der Eerden ME, et al. Integrated palliative care in Europe: a qualitative systematic literature review of empirically-tested models in cancer and chronic disease. BMC Palliative Care 2016;**15**:56.
18. Davidson PM, Paull G, Introna K, et al. Integrated, collaborative palliative care in heart failure: the St. George Heart Failure Service experience 1999-2002. J Cardiovasc Nurs 2004;**19**(1):68-75.
19. Yohannes AM. Palliative care provision for patients with chronic obstructive pulmonary disease. Health Qual Life Outcomes 2007;**5**:17.

For peer review only

For peer review only - http://bmjopen.bmj.com/site/about/guidelines.xhtml

Table 1: Participant characteristics

Characteristic	Number of GPs
**Sex**	
Male	12
Female	13
**Years of experience as GP**	
<5 years	5
5-10 years	10
16-25 years	9
>25 years	1
**Specialist hepatology/gastroenterology experience or training**	
Yes	4
No	21
**Size of practice**	
<5,000	5
5-10,000	9
10,000-15,000	9
>15,000	2
**Geographical area**	
North West London	7
Wessex	8
North East and North Cumbria	5
Yorkshire and Humber	1
Thames Valley and South Midlands	4

For peer review only

For peer review only - http://bmjopen.bmj.com/site/about/guidelines.xhtml

No	Item	Guide questions/description	
**Domain 1: Research team and reflexivity**			
Personal Characteristics			
1.	Interviewer/facilitator	Which author/s conducted the interview or focus group?	HS
2.	Credentials	What were the researcher's credentials? *E.g. PhD, MD*	PhD, MSc, Bsc
3.	Occupation	What was their occupation at the time of the study?	Research Associate in the Institute of Health and Society, Newcastle University
4.	Gender	Was the researcher male or female?	Female
5.	Experience and training	What experience or training did the researcher have?	Conducting interviews and analysing data from patients, clinicians and

No	Item	Guide questions/description	
			family members
Relationship with participants			
6.	Relationship established	Was a relationship established prior to study commencement?	In some areas authors knew the representatives from the clinical research networks who assisted with recruitment. In other areas there was no relationship. There were no prior relationships with GP participants.
7.	Participant knowledge of the	What did the participants know about the researcher?	Reasons for

For peer review only - http://bmjopen.bmj.com/site/about/guidelines.xhtml

No	Item	Guide questions/description	
	interviewer	e.g. *personal goals, reasons for doing the research*	doing the research were provided in information sheets sent to participants before the interviews.
8.	Interviewer characteristics	What characteristics were reported about the interviewer/facilitator? e.g. *Bias, assumptions, reasons and interests in the research topic*	HS is a medical Sociologist with a specialist interest in end of life issues
**Domain 2: study design**			
Theoretical framework			
9.	Methodological orientation and Theory	What methodological orientation was stated to underpin the study? *e.g. grounded theory, discourse analysis, ethnography, phenomenology, content analysis*	Thematic analysis<br>See page 6 of the article

No	Item	Guide questions/description	
Participant selection			
10.	Sampling	How were participants selected? *e.g. purposive, convenience, consecutive, snowball*	Purposive sampling See page 5 of the article
11.	Method of approach	How were participants approached? e.g. *face-to-face, telephone, mail, email*	Face-to-face and telephone See page 5 of the article
12.	Sample size	How many participants were in the study?	25 See page 5 of the article
13.	Non-participation	How many people refused to participate or dropped out? Reasons?	8 GPs expressed interest in participating in the study but later dropped out citing lack of time to participate.

No	Item	Guide questions/description	
Setting			
14.	Setting of data collection	Where was the data collected? e.*g. home, clinic, workplace*	Over the telephone, workplace
15.	Presence of non-participants	Was anyone else present besides the participants and researchers?	No one was present expect the participants and researchers
16.	Description of sample	What are the important characteristics of the sample? *e.g. demographic data, date*	Wide range of clinical experience from GP trainee to >30 years experience Participant characteristics are presented in Table 1
Data collection			

No	Item	Guide questions/description	
17.	Interview guide	Were questions, prompts, guides provided by the authors? Was it pilot tested?	Yes, an interview schedule was developed. This was pilot tested with one interviewee See page 5 of the article
18.	Repeat interviews	Were repeat interviews carried out? If yes, how many?	No repeat interviews were conducted
19.	Audio/visual recording	Did the research use audio or visual recording to collect the data?	Audio recording see page 6 of the article
20.	Field notes	Were field notes made during and/or after the interview or focus group?	Yes see page 6 of the article

No	Item	Guide questions/description	
21.	Duration	What was the duration of the interviews or focus group?	15-50 minutes
22.	Data saturation	Was data saturation discussed?	Yes, data collection was stopped when new themes were no longer emerging from the data. See page 5 of the article
23.	Transcripts returned	Were transcripts returned to participants for comment and/or correction?	No
**Domain 3: analysis and findings**z			
Data analysis			
24.	Number of data coders	How many data coders coded the data?	3 See page 6 of the article

No	Item	Guide questions/description	
25.	Description of the coding tree	Did authors provide a description of the coding tree?	No, documentation detailing development of themes is available upon request.
26.	Derivation of themes	Were themes identified in advance or derived from the data?	Derived from the data<br>See page 6 of the article
27.	Software	What software, if applicable, was used to manage the data?	NVivo10<br>See page 6 of the article
28.	Participant checking	Did participants provide feedback on the findings?	No
Reporting			
29.	Quotations presented	Were participant quotations presented to illustrate the themes / findings? Was each quotation identified? *e.g. participant number*	Yes<br>See pages 7-15 of the article

No	Item	Guide questions/description	
30.	Data and findings consistent	Was there consistency between the data presented and the findings?	Yes there is consistency between the data presented and the findings. See pages 7-15 of the article
31.	Clarity of major themes	Were major themes clearly presented in the findings?	Yes, major themes are presented clearly in the article see pages 7-15.
32.	Clarity of minor themes	Is there a description of diverse cases or discussion of minor themes?	Yes, there are descriptions of diverse and minor themes in the article, see pages 7-15.

BMJ Open

How can primary care enhance end-of-life care for liver disease? A qualitative study of general practitioners' perceptions and experiences

Journal:	*BMJ Open*
Manuscript ID	bmjopen-2017-017106.R1
Article Type:	Research
Date Submitted by the Author:	16-Jun-2017
Complete List of Authors:	Standing, Holly; Newcastle University, Institute of Health & Society<br>Jarvis, Helen; Newcastle University, Institute of Health & Society<br>Orr, James; Newcastle University, Institute of Cellular Medicine<br>Exley, Catherine; Northumbria University, Faculty of Health and Life Sciences<br>Husdon, Mark; Freeman Hospital; Newcastle University, Institute of Cellular Medicine<br>Kaner, Eileen; Newcastle University, Institute of Health and Society<br>Hanratty, Barbara; Newcastle University, Institute of Health and Society / Newcastle University Institute for Ageing
**Primary Subject Heading**:	General practice / Family practice
Secondary Subject Heading:	Gastroenterology and hepatology, Palliative care
Keywords:	PALLIATIVE CARE, liver diseases, terminal care, patient care

SCHOLARONE™
Manuscripts

How can primary care enhance end-of-life care for liver disease? A qualitative study of general practitioners' perceptions and experiences

SUBMISSION TO: BMJ Open

AUTHORS
Holly Standing, Helen Jarvis, James Orr,
Catherine Exley, Mark Hudson, Eileen Kaner, Barbara Hanratty

Dr Holly C Standing
Institute of Health and Society
Newcastle University,
Holly.standing@newcastle.ac.uk

Dr Helen Jarvis
Institute of Health and Society, Newcastle University, Biomedical Research Building, Campus for Ageing and Vitality, Newcastle upon Tyne NE4 5PL
Helen.jarvis2@newcastle.ac.uk

Dr James Orr
Institute of Cellular Medicine, Newcastle University
James.orr@newcastle.ac.uk

Professor Catherine Exley
Faculty of Health and Life Sciences,
Northumbria University,
Newcastle upon Tyne NE1 8ST
Catherine.exley@northumbria.ac.uk

Dr Mark Hudson
Freeman hospital, High Heaton, Newcastle upon Tyne, NE7 7DN
Institute of Cellular Medicine, Newcastle University
Mark.Hudson@nuth.nhs.uk

Professor Eileen Kaner
Institute of Health and Society, Newcastle University, The Baddiley-Clark Building
Richardson Road, Newcastle upon Tyne NE2 4AX
Eileen.kaner@newcastle.ac.uk

Professor Barbara Hanratty*
Institute of Health and Society, Newcastle University, Biomedical Research Building, Campus for Ageing and Vitality, Newcastle upon Tyne NE4 5PL
Barbara.hanratty@newcastle.ac.uk

***Author for correspondence: Barbara Hanratty**

Word count 3941 including quotes)
Key words (MeSH) : terminal care, palliative care, liver diseases, patient care

For peer review only - http://bmjopen.bmj.com/site/about/guidelines.xhtml

Abstract

Background: Liver disease is the third commonest cause of premature death in the UK. The symptoms of terminal liver disease are often difficult to treat, but very few patients see a palliative care specialist, and a high proportion die in hospital. Primary care has been identified as a setting where knowledge and awareness of liver disease is poor. Little is known about general practitioners' (GPs) perceptions of their role in managing end-stage liver disease.

Objective: To explore GPs' experiences and perceptions of how primary care can enhance end-of-life care for patients with liver disease.

Design: Qualitative interview study, thematic analysis

Participants: Purposive sample of 25 GPs from five regions of England

Results: GPs expressed a desire to be more closely involved in end-of-life care for patients with liver disease, but identified a number of factors that constrained their ability to contribute. These fell into three main areas; those relating directly to the condition, (symptom management and the need to combine a palliative care approach with ongoing medical interventions); issues arising from patients' social circumstances (stigma, social isolation and the social consequences of liver disease); and deficiencies in the organisation and delivery of services. Collaborative working with support from specialist hospital clinicians was regarded as essential, with GPs acknowledging their lack of experience and expertise in this area.

Conclusions: End-of-life care for patients with liver disease merits attention from both primary and secondary care services. Development of care pathways and equitable access to symptom relief should be a priority.

For peer review only - http://bmjopen.bmj.com/site/about/guidelines.xhtml

Abstract word count 249

Stength and limitations of this study:

- To the best of our knowledge this is the first study to focus on the experiences' of primary care clinicians around managing end-of-life liver disease.
- GPs were recruited from a range of both rural and urban UK locations.
- Use of Semi-structured interviews allowed us to collect detailed descriptions of GPs' experiences of managing end-of-life liver disease.
- The study was conducted by a team of experienced researchers with a range of disciplinary backgrounds including clinicial experience.
- The findings of our study are based on the reported knowdlege and experiences' of self-selecting participants, their views may not be transferable to the wider GP population.

Introduction

End stage liver disease is an important challenge for providers of palliative and end-of-life care. Death rates have increased 400% since 1970, and it is now the third commonest cause of premature death in the UK.[1, 2 3] In 2012, around 11,000 deaths were attributed to liver disease in the UK. Patients have a complex and heavy symptom burden that is often poorly treated [4 5] and the psychosocial needs of patients and families may be considerable.[6] The majority of patients present late to services, when liver disease is irreversible and around 70% die in hospital.[7] The typical clinical course, of gradual decline punctuated by episodic decompensation, may mean that treatment is focused on prolonging life and a palliative care approach is rarely considered.[8] Transplantation is an option only for selected patients,[9] with

For peer review only - http://bmjopen.bmj.com/site/about/guidelines.xhtml

some evidence suggesting that patients who are considered and rejected for organ transplantation, are unlikely to receive any palliative care.[10] Compared to cancer patients, people with liver disease are less likely to discuss and plan their end of life care.[11] Discussing care plans that acknowledge the proximity of death is difficult, particularly when patients are receiving interventionist care. However, it is important, as timely refereal to palliative care can be compromised when the focus remains inappropriately on treatment with curative intent.[12]

There is a growing international consensus that end-of-life care for patients with liver disease requires improvement.[4 11 13 14] Primary care has been identified as a specific area where care could be enhanced, particularly around the discussion of palliative care needs with patients, the inclusion of patients on palliative care registers, and improving communication between primary and secondary care.[8 15] Primary care professionals are well placed to provide holistic care that patients dying with liver disease need, but are seldom involved. In addition, GP care for patients dying with liver disease is not rated highly by bereaved relatives.[8,16]

Little is known about how health professionals in primary care see their role in end-stage liver disease, or what they view as the main barriers to providing high quality care. This study intends to contribute to our understanding of this patient group and to inform the development of appropriate services. The aim is to explore GP experiences and perceptions of end-of-life care for patients with liver disease.

Methods

This study employed qualitative methods, involving semi-structured interviews with GPs from five geographical areas within England.

For peer review only - http://bmjopen.bmj.com/site/about/guidelines.xhtml

Participants

Participants were recruited via National Institute for Health Research Clinical Research Networks and local networks of GP practices in the London, Thames Valley, Wessex, Yorkshire and the North East. Email invitations were sent to GP practices within these networks, and GPs who wished to participate then contacted the research team. Following the first phase of interviews, participants were purposively sampled in order to provide a wide range of clinical experience and degree of familiarity with liver disease. To do this, we expanded the study to include two additional georgraphical sites and worked with co-ordinators at the Clinical Research Networks to target practices in a variety of rural and urban locations, as well as areas of high and low prevalence of liver disease and substance misuse.

Data collection

A semi-structured interview guide was developed to cover issues identified through a review of the literature; including GPs' experiences of identifying and managing end-of-life liver disease. This was a 'living' document that evolved throughout data collection to allow exploration of emerging areas. Interviews were conducted face-to-face (n=2) or over the telephone (n=23). Interviews lasted between 15 and 50 minutes and were all conducted by the first author between March and August 2016. Field notes were taken to aid subsequent analysis. Informed consent was obtained from all participants. Data collection ceased when no themes were emerging from the interviews (see below for further detail).

For peer review only - http://bmjopen.bmj.com/site/about/guidelines.xhtml

Data analysis

Audio recordings of interviews were transcribed verbatim by an independent transcription company; transcripts were checked for accuracy by listening again to each recording. The NVivo 10 software package was used to manage the data.

Data collection and analysis ran concurrently throughout the study. Analysis of early transcripts informed the interview schedule for later interviews and each transcript was re-examined in light of subsequent interviews. A thematic analysis was conducted.[17] The first stage involved researchers familiarising themselves with the data through detailed reading of the transcripts followed by line-by-line coding.[18] Field notes taken during data collection were used throughout analysis to enhance the reflective process. Several quality control measures were employed to ensure trustworthiness of the data. A proportion of the transcripts (20%) were coded independently by three researchers, before coming together to compare their analysis. Data analysis and emerging themes were also discussed amongst the wider research team, which included individuals with clinical expertise in general practice and hepatology.

Findings

Twenty five GPs were interviewed. The majority had been qualified as GPs for five or more years, but few (4/25) had any specialist hepatology or gastroenterology training or experience. Participant characteristics are shown in table 1.

For peer review only - http://bmjopen.bmj.com/site/about/guidelines.xhtml

Table 1 here

Four themes were identified from the data analysis: the role of the GP, acknowledging and accepting end-of-life, collaborative care pathways, and social relationships and consequences. The quotations presented below are illustrative, representing typical participant responses and demonstrating the varied viewpoints.

For peer review only

The role of the GP

In this study, few of the interviewees had extensive first-hand experience of managing patients with liver disease at the end-of-life. Those who did, reported that they managed such cases infrequently, and some years may go by without them seeing a case.

> *"[We manage] a lot of dying people, but not from the hepatology point of view. I don't know if they tend to be managed in hospital predominantly more than in primary care? That's a possibility, I guess."* (GP 7)

Some of the interviewees attributed their lack of expertise and experience of caring for liver patients at the end-of-life to a reluctance amongst hospital clinicians to relinquish control.

> *"There are some conditions, like liver disease, renal failure, they [patients] all just end up dying in hospital for some reason. I don't know whether it's the hospital consultants that don't want to let them*

For peer review only - http://bmjopen.bmj.com/site/about/guidelines.xhtml

go home… They need to let go and make sure there's a palliative care plan in place…they don't do it." (GP 3)

The limited contact between GPs and patients dying with liver disease was attributed to an unpredictable disease trajectory with periods of stability and decompensation, and to patients remaining under the care of hospital services in their last weeks and months. The GPs in this study shared a view that end-of-life care is a core component of primary care, and interviewees questioned how appropriate it was for specialist hospital clinicians to take a lead in palliative care. Patients with liver disease were not regarded as distinct or different from patients dying with other conditions, and a number of GPs expressed a desire for greater involvement in their end-of-life care. Some participants implied that primary care involvement may support more patients to die at home, rather than in hospital.

"I think primary care probably is best placed, in most cases, to look after people- well not only for that [liver disease], for most end-of-life care issues. So, yeah, I think the GP is probably the most important person in the sense that they can bear in mind what the specialists have advised, but at the end of the day, try and keep some of these patients at home rather than having to have them admitted acutely." (GP 14)

Acknowledging and accepting end-of-life

Judging when a patient with liver disease is nearing the end-of-life was perceived to be a particular challenge. Communication about prognosis and the age of patients

For peer review only

For peer review only - http://bmjopen.bmj.com/site/about/guidelines.xhtml

were identified as important factors. Some of the GPs reflected on how management decisions taken in hospital send out messages that influence care provided in the community. Continuing to pursue active treatment may convey optimism about the patient's life expectancy. Specifically, GPs referenced occasions where patients had been placed on the waiting list for a liver transplant, which the patient saw as offering them a second chance at life, even though they were critically unwell and may die whilst waiting for an organ. Patients with end stage liver disease are often younger than the typical palliative care patient.[19] It may be that clinicians are more reluctant to give up on active treatment for younger patients,[20] whilst patients and families may also struggle to accept that the end-of-life is approaching.[21]

> *"Those patients where it's a, kind of, grey area about whether they're end-of-life or not. And I think that mainly stems from the fact that if it's a young patient, it's more difficult for health care professionals, the patients themselves, and families, to actually accept that the person's dying"* (GP1)

Mixed or uncertain messages may mean that care is compromised, if no one engages the patient in discussions about the end-of-life, and a palliative approach is never considered.

> *"I suppose, looking back it really was palliative care but they [secondary care] put him on the transplant list because he's given up alcohol and there was still this hope. So therefore we didn't really*

For peer review only - http://bmjopen.bmj.com/site/about/guidelines.xhtml

realise he was going to die as quickly as he did" (GP 11)

There was a shared feeling amongst interviewees, that specialists should provide clear messages about patients' prognoses, so that GPs can adopt an appropriate management plan. At present, hospital specialists were perceived as failing to take responsibility for identifying patients as end-of-life, and this had a detrimental impact on primary care.

> *"I feel that it should be made compulsory for the secondary care, tertiary care sectors, when they discharge, or when they're seen in the patient clinic, [to] prognosticate, … then we can initiate also, the discussion with the patient, in a much more positive way."* (GP 12)

Collaborative care pathways

Supporting patients with liver disease was seen as a collaborative effort, with GPs acknowledging their need for specialist guidance, particularly when managing end-of-life complications. A small number of respondents mentioned hepatic encephalopathy as a challenge in the management of end-of-life liver patients, and a potential source of distress for relatives. The interviewees suggested that they would benefit from further training to deal with this complication. Ascites was the most commonly mentioned symptom experienced by patients with end-stage liver disease, requiring drainage in hospital. Experiences of GPs in this study suggest that ease of access to this procedure was highly variable. In some areas, pathways had been negotiated and patients could be directly admitted to an appropriate ward. In others, GPs described their concern at having to send patients to accident and

For peer review only - http://bmjopen.bmj.com/site/about/guidelines.xhtml

For peer review only

emergency (A&E) departments. Failure to arrange prompt access to treatment caused distress and was a major source of adverse experiences during end-of-life care.

> *"We had a nightmare. He was building up litres of ascitic fluid on his tummy every week or week to 10 days, and every time the hospital had to send him acutely, new, to A&E and he had to sit in A&E for hours. I was speaking to the liver specialist ... He needed regular reviews and eventually they agreed to do it two weekly but even that wasn't enough, it was building up and he was ending up going in as an emergency every week."* (GP 11)

Where appropriate care pathways were not in place, interviewees suggested that they were needed, to reassure the patient and GPs that support is available when required.

A number of participants suggested that a specialist nurse may hold the key to more collaborative management of liver patients. They could act as an intermediary between primary and secondary care, negotiating priorities and ensuring effective and easy communication.

> *"It often helps when there is direct access to, say, a nurse specialist in a field, or there is some other point of contact in secondary care that say a family or the patient themselves can call directly for advice."* (GP 14)

Although some GPs had encountered specialist nurses working in this type of role, this was not a common experience. Unfavourable comparisons were made between

For peer review only - http://bmjopen.bmj.com/site/about/guidelines.xhtml

the services available for patients with liver disease, and other conditions, such as cancer. Participants highlighted the potential benefit to patients and families, of having a specialist point of contact in the community, including prompt access to advice and alleviation of fears and concerns.

Social relationships and consequences

GPs in this study argued that people with liver disease had many of the same primary care needs as patients with other life-limiting conditions. However, the severity of symptoms in end-of-life liver disease was felt to be different. Some of the GPs acknowledged the potentially damaging impact on the patient's family, of seeing their relative die at home.

> *"I think there is quite a strong push to keep people at home. Whether that's right or wrong, I don't know really. If they've got ascites or portal hypertension, you know, they've got the risk of vomiting blood and all the rest of it. Or they have been vomiting blood. I'm not massively keen on keeping people at home because it's just a rubbish picture in the mind of everybody, I think, you know, the family left behind."* (GP 17)

Families were perceived to be in need of support themselves, which was an additional role for primary care. GPs described examples of relatives requiring frequent contact and reassurance as the patient's condition deteriorated. The GPs in this study differed in their attitudes towards these demands. Some took a holistic

For peer review only - http://bmjopen.bmj.com/site/about/guidelines.xhtml

For peer review only

view to the management of palliative patients, believing that these were part of the standard practice of primary care.

> *"I think when we talk about palliative care it's not just a single person who's the patient, it's about supporting and managing the family and helping them through that bereavement stage because it starts right at the diagnosis and they have to go through that journey. Death is a part of life and giving them that support."* (GP 10)

However, others felt that attending to the needs of patients' families was an extra burden on their already overstretched resources.

Limited social support and unfavourable social circumstances were often mentioned as significant issues for patients with liver disease, particularly when alcohol or drug misuse were factors. Several GPs referred to the 'chaotic' lifestyles of this patient group, and resulting vulnerability to social isolation. Behaviours associated with addiction were perceived to lead to the breakdown of the patients' social networks, leaving few, if any people to provide support or care.

> *"the demographics of the alcohol dependent ones, who have often, for various reasons and due to the nature of their disease, have become quite isolated, they have not got many people around them and so they don't have that support. They require much more organisation and support in the background, so we make sure that they do have that support."* (GP 10)

For peer review only

For peer review only - http://bmjopen.bmj.com/site/about/guidelines.xhtml

Without alternative sources of support, socially isolated patients were believed to place extra demands on GPs and other health services. Even when social networks were maintained, there could be a dearth of responsible caregivers, as friends and family often shared the problems of addiction and poor health.

> *"I can think of a couple of our households where maybe spouses and partners may have liver cirrhosis themselves. I can think of two couples – well, one person who died two years ago. His wife has chronic liver disease as well."* (GP 4)

One of the most important consequences of social isolation, was that patients had fewer choices over where they spent the end of their life. Without anyone to monitor their condition, they were more likely to be admitted to hospital, and die there.

Liver disease is a potentially stigmatising condition, particularly when the underlying cause is alcohol or substance misuse. Several of the GPs suggested that there is often an assumption within the patient's community that liver disease is self-induced and they were culpable, even when substance misuse or alcohol are not factors.

> *"I think it's a huge problem for people that have liver disease and look like they have liver disease and people assume it's related to alcohol when, in fact, it might be due to auto-immune causes or other forms of cancer or something like that or hepatitis as well."* (GP 23)

For peer review only - http://bmjopen.bmj.com/site/about/guidelines.xhtml

This assumed culpability has implications for the degree of support and sympathy that the patient, and their families, receive. GPs also suggested that stigma could hinder patients' acceptance of their prognosis, which in turn made the management of their condition more challenging. As such, care of liver patients should include psychological and social services.

> *"I think, inevitably and sadly, there is a stigma associated with liver disease, and hence, that's why the psychological support is really important"* (GP 25)

However, some commented that stigmatisation occurred early in the patients' illnesses, and to address this, changes would be needed well before end-of-life care was being considered.

Discussion

This study provides insights into the challenge faced by general practitioners providing end-of-life care for patients with chronic liver disease. Many GPs expressed a desire to be more closely involved, but identified a number of factors that constrained their ability to contribute. These fell into three main areas; those relating directly to the condition, (symptom management and the need to combine a palliative care approach with ongoing medical interventions); issues arising from patients' social circumstances (stigma, social isolation and the social consequences of liver disease); and deficiencies in the organisation and delivery of services. Collaborative working with support from specialist hospital clinicians was regarded as essential, with GPs acknowledging their lack of experience and expertise in this area.

For peer review only - http://bmjopen.bmj.com/site/about/guidelines.xhtml

A majority of interviewees had little direct experience of patients dying of liver disease and as a consequence, they may not have been familiar with all the management challenges of end stage liver disease. For example, hepatic encephalopathy is a common concern in the care of end stage liver patients, yet few of the interviewees discussed it. This is not surprising, as primary care clinicians would seldom have responsibility for managing hepatic encephalopathy, if they are not dealing day to day with end-of-life care for liver disease patients.

Strengths and limitations

To our knowledge this is the first study to focus on the experiences of primary care physicians in managing patients with end-stage liver disease. Our interviewees were drawn from rural and urban areas in five different regions in England, and working with a diverse range of communities. The relatively large number of GP participants and varying levels of experience, expertise and interest in the subject, is a particular strength of the study. With our qualitative design, we were not seeking generalisability, but the diversity of the participants increases our confidence that we have not overlooked important issues.

The majority of interviews were conducted by telephone, which may explain the ease and speed with which we recruited participants, despite not offering any financial incentives. Use of the telephone is thought to have promoted unguarded responses, but we acknowledge that it can be more difficult to develop rapport in the absence of nonverbal cues and other facets of face to face communication. However, we do not

For peer review only - http://bmjopen.bmj.com/site/about/guidelines.xhtml

For peer review only

believe that this was a problem, as GPs provided rich and insightful accounts of their experiences.

Comparison with other work

Our findings are consistent with recent research from Scotland that included interviews with eight GPs along with other health care professionals. Communication with secondary care, lack of expertise and limited confidence in prognostication were all identified as concerns.[15] Accurate assessment of prognosis in liver disease is difficult given the unpredictable disease course. In some aspects this is similar to other diseases characterised by episodes of decompensation, such as heart failure. However, liver disease presents the additional challenge that recompensation and improved liver function may be achieved in certain patients, such as those who achieve abstinence from alcohol. A recent review of palliative care guidelines in heart failure and chronic obstructive pulmonary disease described wide variation in how patients are identified for palliative care, and attributed this, in part, to the unpredictable disease course and the consequences for care planning.[22] In common with liver disease, acknowledgement and development of end-of-life care has been relatively recent for these conditions.[22]

The GPs in our study agreed that, at the end-of-life, patients with liver disease ideally need primary care and hospital specialists to work closely together. GPs are more likely to have an established relationship with the patient and a greater understanding of their social situation and needs, whereas specialists offer expert knowledge on liver disease and treatment options. They highlighted the importance that primary care physicians place on being able to provide a coordinating role, but

For peer review only - http://bmjopen.bmj.com/site/about/guidelines.xhtml

For peer review only

only when supported by members of the specialist teams. Managing complex and unusual symptoms, or judging when to introduce a palliative care approach, for example, all benefit from collaboration. The advantages of a multidisciplinary approach have already been well documented in the palliative care literature.[23 24] Several recent reviews on end-stage liver disease have also advocated this approach.[4 5 14]

This study highlights the complexity of caring for patients with end-stage liver disease. Expertise in acute medicine and palliative care are essential, but patients and families also need sensitive and practical responses to their psycho-emotional and social concerns, including stigma related to the perceived self-inflictedness of the disease, social isolation and lack of income. Such generalist expertise and a holistic, person-centred approach are the foundations of primary care. Community-based services already play an important role at the end-of-life for patients with many different, complex conditions. However, this seldom includes people dying with liver disease. Greater involvement of community services would be expected to enhance the quality and appropriateness of palliative and terminal care for these patients. As the number of deaths from chronic liver disease increases, it may be increasingly necessary, in order to limit the burden on hospital teams. Innovations, such as the development of clear patient pathways, specialist heptology nurses in the community or district nurses trained to deal with liver disease complications, all require resources. Specialist treatments such as paracentesis could be delivered in locations such as community hospitals or hospices, where they are available, to reduce disruption to patients' lives.

(Box 1 here)

For peer review only - http://bmjopen.bmj.com/site/about/guidelines.xhtml

In recent years, UK health policy has increasingly promoted patient choice; an ability to deliver end of life care in the patient's preferred location and facilitate choice in place of death are used as markers of care quality, with death at home often an implicit goal of palliative care.[25 26] GPs in this study expressed some scepticism that home death is always the best option for patients with liver disease, or their families. Concerns centred around the nature of the symptoms and clinical input needed to manage them, which were potentially distressing for families to observe. Balancing the wishes of patients, families and clinical carers is a fundamental part of end of life care. More in depth enquiry to elicit patient, family and professional views and experiences of place of death in liver disease would help to clarify the resources required to ensure death at home is acceptable and achievable.

Conclusion

Our study suggests that end-of-life care for patients with liver disease requires attention. Liver disease appears to pose management challenges in end-of-life care with a combination of complicated social situations and symptoms. Services tailored for these patients should build on the similarities with other conditions, but also reflect the differences. The adverse social consequences of illness for these patients and their families, may be particularly significant. Further research is needed to fully understand the burden on families and services. As health services seek greater integration with social care, improving care for patients with end-stage liver disease should be a priority.

For peer review only - http://bmjopen.bmj.com/site/about/guidelines.xhtml

Acknowledgements

We are grateful to the participants who generously gave their time to this study.

Ethics Approval

This study involved no patients and was assessed by the Health Research Authority and Newcastle University Research Ethics (Ref 188275).

Authors contribution

BH, EK, MH, JO, and CE designed the study. HS carried out the interviews. HS undertook the main analysis supported by BH and HJ. HS, HJ and BH drafted the manuscript, and all authors commented and approved the final version.

Funding statement

This work was supported by the NIHR School for Primary Care grant number 262.

Competing interests

We have read and understood BMJ policy on declaration of interests and declare that we have no competing interests.

Data sharing agreement

We do not have any additional unpublished data that can be shared.

References

1. World Health Organisation. WHO European health for all database. 2012. http://data.euro.who.int/ (accessed 22 January 2017).
2. Office for National Statistics. Deaths registered in England and Wales: 2015. 2016; (9th September 2016). https://www.ons.gov.uk/peoplepopulationandcommunity/birthsdeathsandmarriages/deaths/bulletins/deathsregistrationsummarytables/2015 (accessed 22 January 2017).
3. University of Washington. The Liver Imaging Atlas: An Online Reference for Liver Imaging. 2017; 2017(22 January 2017). liveratlas.org (accessed 22 January 2017).
4. Boyd K, Kimbell B, Murray S, et al. Living and dying well with end-stage liver disease: Time for palliative care? *Hepatology* 2012;55(6):1650-51. doi: 10.1002/hep.25621
5. Roth K, Lynn J, Zhong Z, et al. Dying with end stage liver disease with cirrhosis: insights from SUPPORT. Study to Understand Prognoses and Preferences for Outcomes and Risks of Treatment. *Journal of the American Geriatrics Society* 2000;48(5 Suppl):S122-30.
6. Kimbell B, Murray SA. What is the patient experience in advanced liver disease? A scoping review of the literature. *BMJ Supportive & Palliative Care* 2013;bmjspcare-2012 doi: 10.1136/bmjspcare-2012-000435

For peer review only - http://bmjopen.bmj.com/site/about/guidelines.xhtml

7. National End of Life Care Intelligence Network. Deaths from Liver Disease: Implications for end of life care in England. 2012. http://www.endoflifecare-intelligence.org.uk/resources/publications/deaths_from_liver_disease (accessed 27 Feburary 2017).
8. Kendrick E. Getting it Right Improving End of Life Care for People Living with Liver Disease. London: National End of Life Care Programme, 2013.
9. European Association for the Study of the Liver. EASL clinicial practice guidelines: liver transplantation. *Journal of Hepatology* 2016;64(2):433-85.
10. Poonja Z, Brisebois A, van Zanten SV, et al. Patients with cirrhosis and denied liver transplants rarely receive adequate palliative care or appropriate management. *Clinical Gastroenterology and Hepatology: The Official Clinical Practice Journal of the American Gastroenterological Association* 2014;12(4):692-98. doi: 10.1016/j.cgh.2013.08.027
11. Potosek J, Curry M, Buss M, et al. Integration of Palliative Care in End-Stage Liver Disease and Liver Transplantation. *Journal of Palliative Medicine* 2014;17(11):1271-77.
12. Gott M IC, Gardiner C, Richards N, Cobb M, Ryan A, Noble B, Bennett M, Seymour J, Ward S, Parker C. Transitions to palliative care for older people in acute hospitals: a mixed-methods study. . *Health Services and Delivery Research* 2013;1(11)
13. Larson AM. Palliative Care for Patients with End-Stage Liver Disease. *Current Gastroenterology Reports* 2015;17(5):18. doi: 10.1007/s11894-015-0440-6
14. Walling AM, Wenger NS. Palliative care and end-stage liver disease. *Clinical Gastroenterology and Hepatology: The Official Clinical Practice Journal of the American Gastroenterological Association* 2014;12(4):699-700. doi: 10.1016/j.cgh.2013.11.010
15. Kimbell B, Boyd K, Kendall M, et al. Managing uncertainty in advanced liver disease: a qualitative, multiperspective, serial interview study. *BMJ open* 2015;5(11):e009241. doi: 10.1136/bmjopen-2015-009241
16. Office for National Statistics. National Survey of Bereaved People (VOICES). 2015. (accessed 27 Feburary 2016).
17. Braun V, Clarke, V. Using thematic analysis in psychology. *Qualitative Research in Psychology* 2006;3(2):77-101. doi: 10.1191/1478088706qp063oa
18. Ritche J, Lewis J. Qualitative Research Practie: A Guide for Social Science Students and Researchers. London: SAGE Publications 2003.
19. Office for National Statistics. Deaths registered in England and Wales (Series DR): 2015. 2016. (accessed 31 May 2017).
20. Department of Health. The impact of patient age on clinical decision-making in oncology. 2012.
21. Thompson GN, Chochinov, H.M., Wilson, K. G., McPherson, C. J., Chary, S., O'Shea, F. M., Kuhl, D. R., Fainsinger, R. L., Gagnon, P.R., Macmillan, K. A. Prognostic Acceptance and the Well-Being of Patients Receiving Palliative Care for Cancer. *Journal of Clinical Oncology* 2009;27(34):5757-62.
22. Siouta N, Van Beek K, van der Eerden ME, et al. Integrated palliative care in Europe: a qualitative systematic literature review of empirically-tested models in cancer and chronic disease. *BMC Palliative Care* 2016;15:56. doi: 10.1186/s12904-016-0130-7
23. Davidson PM, Paull G, Introna K, et al. Integrated, collaborative palliative care in heart failure: the St. George Heart Failure Service experience 1999-2002. *The Journal of Cardiovascular Nursing* 2004;19(1):68-75.
24. Yohannes AM. Palliative care provision for patients with chronic obstructive pulmonary disease. *Health and Quality of Life Outcomes* 2007;5:17. doi: 10.1186/1477-7525-5-17
25. Department of Health. End of Life Care Strategy. 2008. https://www.gov.uk/government/publications/end-of-life-care-strategy-promoting-high-quality-care-for-adults-at-the-end-of-their-life (accessed 13th June 2017).
26. National Institute for Health and Care Excellence. NICE Quality Standards on End of Life Care. 2011. https://www.nice.org.uk/guidance/qs13 (accessed 13th June 2017).

For peer review only - http://bmjopen.bmj.com/site/about/guidelines.xhtml

Table 1: Participant characteristics

Characteristic	Number of GPs
**Sex**	
Male	12
Female	13
**Years of experience as GP**	
<5 years	5
5-10 years	10
16-25 years	9
>25 years	1
**Specialist hepatology/gastroenterology experience or training**	
Yes	4
No	21
**Size of practice**	
<5,000	5
5-10,000	9
10,000-15,000	9
>15,000	2
**Geographical area**	
North West London	7
Wessex	8
North East and North Cumbria	5
Yorkshire and Humber	1
Thames Valley and South Midlands	4

Box 1

Next steps in primary end of life care for liver disease: GP perceptions of areas for development

- Education and training in symptom management for end stage liver diease
- Collaborative care pathways between primary care and hepatologists
- Service delivery that takes into account patient and family social circumstances and stigma
- Provision of support for family caregivers
- Consideration / research into the role of home death

For peer review only

For peer review only

For peer review only - http://bmjopen.bmj.com/site/about/guidelines.xhtml

No	Item	Guide questions/description	
**Domain 1: Research team and reflexivity**			
Personal Characteristics			
1.	Interviewer/facilitator	Which author/s conducted the interview or focus group?	HS
2.	Credentials	What were the researcher's credentials? *E.g. PhD, MD*	PhD, MSc, Bsc
3.	Occupation	What was their occupation at the time of the study?	Research Associate in the Institute of Health and Society, Newcastle University
4.	Gender	Was the researcher male or female?	Female
5.	Experience and training	What experience or training did the researcher have?	Conducting interviews and analysing data from patients, clinicians and

No	Item	Guide questions/description	
			family members
Relationship with participants			
6.	Relationship established	Was a relationship established prior to study commencement?	In some areas authors knew the representatives from the clinical research networks who assisted with recruitment. In other areas there was no relationship. There were no prior relationships with GP participants.
7.	Participant knowledge of the	What did the participants know about the researcher?	Reasons for

No	Item	Guide questions/description	
	interviewer	e.g. *personal goals, reasons for doing the research*	doing the research were provided in information sheets sent to participants before the interviews.
8.	Interviewer characteristics	What characteristics were reported about the interviewer/facilitator? e.g. *Bias, assumptions, reasons and interests in the research topic*	HS is a medical Sociologist with a specialist interest in end of life issues
**Domain 2: study design**			
Theoretical framework			
9.	Methodological orientation and Theory	What methodological orientation was stated to underpin the study? *e.g. grounded theory, discourse analysis, ethnography, phenomenology, content analysis*	Thematic analysis See page 6 of the article

No	Item	Guide questions/description	
Participant selection			
10.	Sampling	How were participants selected? *e.g. purposive, convenience, consecutive, snowball*	Purposive sampling<br>See page 5 of the article
11.	Method of approach	How were participants approached? e.g. *face-to-face, telephone, mail, email*	Face-to-face and telephone<br>See page 5 of the article
12.	Sample size	How many participants were in the study?	25<br>See page 5 of the article
13.	Non-participation	How many people refused to participate or dropped out? Reasons?	8 GPs expressed interest in participating in the study but later dropped out citing lack of time to participate.

No	Item	Guide questions/description	
Setting			
14.	Setting of data collection	Where was the data collected? e.g. *home, clinic, workplace*	Over the telephone, workplace
15.	Presence of non-participants	Was anyone else present besides the participants and researchers?	No one was present expect the participants and researchers
16.	Description of sample	What are the important characteristics of the sample? *e.g. demographic data, date*	Wide range of clinical experience from GP trainee to >30 years experience Participant characteristics are presented in Table 1
Data collection			

No	Item	Guide questions/description	
17.	Interview guide	Were questions, prompts, guides provided by the authors? Was it pilot tested?	Yes, an interview schedule was developed. This was pilot tested with one interviewee See page 5 of the article
18.	Repeat interviews	Were repeat interviews carried out? If yes, how many?	No repeat interviews were conducted
19.	Audio/visual recording	Did the research use audio or visual recording to collect the data?	Audio recording see page 6 of the article
20.	Field notes	Were field notes made during and/or after the interview or focus group?	Yes see page 6 of the article

No	Item	Guide questions/description	
21.	Duration	What was the duration of the interviews or focus group?	15-50 minutes
22.	Data saturation	Was data saturation discussed?	Yes, data collection was stopped when new themes were no longer emerging from the data.<br>See page 5 of the article
23.	Transcripts returned	Were transcripts returned to participants for comment and/or correction?	No
**Domain 3: analysis and findings**z			
Data analysis			
24.	Number of data coders	How many data coders coded the data?	3<br>See page 6 of the article

No	Item	Guide questions/description	
25.	Description of the coding tree	Did authors provide a description of the coding tree?	No, documentation detailing development of themes is available upon request.
26.	Derivation of themes	Were themes identified in advance or derived from the data?	Derived from the data<br>See page 6 of the article
27.	Software	What software, if applicable, was used to manage the data?	NVivo10<br>See page 6 of the article
28.	Participant checking	Did participants provide feedback on the findings?	No
Reporting			
29.	Quotations presented	Were participant quotations presented to illustrate the themes / findings? Was each quotation identified? e.g. *participant number*	Yes<br>See pages 7-15 of the article

No	Item	Guide questions/description	
30.	Data and findings consistent	Was there consistency between the data presented and the findings?	Yes there is consistency between the data presented and the findings. See pages 7-15 of the article
31.	Clarity of major themes	Were major themes clearly presented in the findings?	Yes, major themes are presented clearly in the article see pages 7-15.
32.	Clarity of minor themes	Is there a description of diverse cases or discussion of minor themes?	Yes, there are descriptions of diverse and minor themes in the article, see pages 7-15.

BMJ Open

How can primary care enhance end-of-life care for liver disease? A qualitative study of general practitioners' perceptions and experiences

Journal:	*BMJ Open*
Manuscript ID	bmjopen-2017-017106.R2
Article Type:	Research
Date Submitted by the Author:	13-Jul-2017
Complete List of Authors:	Standing, Holly; Newcastle University, Institute of Health & Society<br>Jarvis, Helen; Newcastle University, Institute of Health & Society<br>Orr, James; Newcastle University, Institute of Cellular Medicine<br>Exley, Catherine; Northumbria University, Faculty of Health and Life Sciences<br>Husdon, Mark; Freeman Hospital; Newcastle University, Institute of Cellular Medicine<br>Kaner, Eileen; Newcastle University, Institute of Health and Society<br>Hanratty, Barbara; Newcastle University, Institute of Health and Society / Newcastle University Institute for Ageing
**Primary Subject Heading**:	General practice / Family practice
Secondary Subject Heading:	Gastroenterology and hepatology, Palliative care
Keywords:	PALLIATIVE CARE, liver diseases, terminal care, patient care

SCHOLARONE™ Manuscripts

For peer review only

How can primary care enhance end-of-life care for liver disease? A qualitative study of general practitioners' perceptions and experiences

SUBMISSION TO: BMJ Open

AUTHORS
Holly Standing, Helen Jarvis, James Orr,
Catherine Exley, Mark Hudson, Eileen Kaner, Barbara Hanratty

Dr Holly C Standing
Institute of Health and Society
Newcastle University,
Holly.standing@newcastle.ac.uk

Dr Helen Jarvis
Institute of Health and Society, Newcastle University, Biomedical Research Building, Campus for Ageing and Vitality, Newcastle upon Tyne NE4 5PL
Helen.jarvis2@newcastle.ac.uk

Dr James Orr
Institute of Cellular Medicine, Newcastle University
James.orr@newcastle.ac.uk

Professor Catherine Exley
Faculty of Health and Life Sciences,
Northumbria University,
Newcastle upon Tyne NE1 8ST
Catherine.exley@northumbria.ac.uk

Dr Mark Hudson
Freeman hospital, High Heaton, Newcastle upon Tyne, NE7 7DN
Institute of Cellular Medicine, Newcastle University
Mark.Hudson@nuth.nhs.uk

Professor Eileen Kaner
Institute of Health and Society, Newcastle University, The Baddiley-Clark Building
Richardson Road, Newcastle upon Tyne NE2 4AX
Eileen.kaner@newcastle.ac.uk

Professor Barbara Hanratty*
Institute of Health and Society, Newcastle University, Biomedical Research Building, Campus for Ageing and Vitality, Newcastle upon Tyne NE4 5PL
Barbara.hanratty@newcastle.ac.uk

***Author for correspondence: Barbara Hanratty**

Word count 4091 including quotes)
Key words (MeSH) : terminal care, palliative care, liver diseases, patient care

For peer review only - http://bmjopen.bmj.com/site/about/guidelines.xhtml

For peer review only

Abstract

Background: Liver disease is the third commonest cause of premature death in the UK. The symptoms of terminal liver disease are often difficult to treat, but very few patients see a palliative care specialist, and a high proportion die in hospital. Primary care has been identified as a setting where knowledge and awareness of liver disease is poor. Little is known about general practitioners' (GPs) perceptions of their role in managing end-stage liver disease.

Objective: To explore GPs' experiences and perceptions of how primary care can enhance end-of-life care for patients with liver disease.

Design: Qualitative interview study, thematic analysis

Participants: Purposive sample of 25 GPs from five regions of England

Results: GPs expressed a desire to be more closely involved in end-of-life care for patients with liver disease, but identified a number of factors that constrained their ability to contribute. These fell into three main areas; those relating directly to the condition, (symptom management and the need to combine a palliative care approach with ongoing medical interventions); issues arising from patients' social circumstances (stigma, social isolation and the social consequences of liver disease); and deficiencies in the organisation and delivery of services. Collaborative working with support from specialist hospital clinicians was regarded as essential, with GPs acknowledging their lack of experience and expertise in this area.

Conclusions: End-of-life care for patients with liver disease merits attention from both primary and secondary care services. Development of care pathways and equitable access to symptom relief should be a priority.

For peer review only - http://bmjopen.bmj.com/site/about/guidelines.xhtml

Abstract word count 249

Stength and limitations of this study:

- To the best of our knowledge this is the first study to focus on the experiences' of primary care clinicians around managing end-of-life liver disease.
- GPs were recruited from a range of both rural and urban UK locations.
- Use of Semi-structured interviews allowed us to collect detailed descriptions of GPs' experiences of managing end-of-life liver disease.
- The study was conducted by a team of experienced researchers with a range of disciplinary backgrounds including clinicial experience.
- The findings of our study are based on the reported knowdlege and experiences' of self-selecting participants, their views may not be transferable to the wider GP population.

Introduction

End stage liver disease is an important challenge for providers of palliative and end-of-life care. Death rates have increased 400% since 1970, and it is now the third commonest cause of premature death in the UK.[1, 2 3] In 2012, around 11,000 deaths were attributed to liver disease in the UK. Patients have a complex and heavy symptom burden that is often poorly treated [4 5] and the psychosocial needs of patients and families may be considerable.[6] The majority of patients present late to services, when liver disease is irreversible and around 70% die in hospital.[7] The typical clinical course, of gradual decline punctuated by episodic decompensation, may mean that treatment is focused on prolonging life and a palliative care approach is rarely considered.[8] Transplantation is an option only for selected patients,[9] with

For peer review only - http://bmjopen.bmj.com/site/about/guidelines.xhtml
For peer review only

some evidence suggesting that patients who are considered and rejected for organ transplantation, are unlikely to receive any palliative care.[10] Research suggests that people with liver disease are less likely to be involved in end of life discussions and planning than cancer patients, though data are limited .[11] Discussing care plans that acknowledge the proximity of death is difficult, particularly when patients are receiving interventionist care. However, it is important, as timely refereal to palliative care can be compromised when the focus remains inappropriately on treatment with curative intent.[12]

There is a growing international consensus that end-of-life care for patients with liver disease requires improvement.[4 11 13 14] Primary care has been identified as a specific area where care could be enhanced, particularly around the discussion of palliative care needs with patients, the inclusion of patients on palliative care registers, and improving communication between primary and secondary care.[8 15] Primary care professionals are well placed to provide holistic care that patients dying with liver disease need, but are seldom involved. In addition, GP care for patients dying with liver disease is not rated highly by bereaved relatives.[8,16]

Little is known about how health professionals in primary care see their role in end-stage liver disease, or what they view as the main barriers to providing high quality care. This study intends to contribute to our understanding of this patient group and to inform the development of appropriate services. The aim is to explore GP experiences and perceptions of end-of-life care for patients with liver disease.

For peer review only - http://bmjopen.bmj.com/site/about/guidelines.xhtml

Methods

This study employed qualitative methods, involving semi-structured interviews with GPs from five geographical areas within England.

Participants

A heterogeneous purposive sampling approach was employed to ensure that a variety of perspectives and experiences of management of liver disease were sampled e.g. previous management of an end of life liver patient, views on whether management should be primary care or secondary care led, as well as a range of practice sizes and localities. Participants were recruited via National Institute for Health Research Clinical Research Networks (CRN) and local networks of GP practices in London, Thames Valley, Wessex, Yorkshire and the North East of England. Sampling began with one CRN and was expanded during the course of the study to include four additional areas. Co-ordinators at the CRNs were utilised to target practices in a variety of rural and urban locations, as well as areas of high and low prevalence of liver disease and substance misuse. Email invitations were sent to GP practices within these networks, and GPs who wished to participate then contacted the research team..

Data collection

A semi-structured interview guide was developed to cover issues identified through a review of the literature; including GPs' experiences of identifying and managing end-of-life liver disease. This was a 'living' document that evolved throughout data collection to allow exploration of emerging areas. Interviews were conducted face-to-face (n=2) or over the telephone (n=23). Interviews lasted between 15 and 50

For peer review only - http://bmjopen.bmj.com/site/about/guidelines.xhtml

minutes and were all conducted by the first author between March and August 2016. Field notes were taken to aid subsequent analysis. Informed consent was obtained from all participants. Data collection ceased when no themes were emerging from the interviews (see below for further detail).

Data analysis

Audio recordings of interviews were transcribed verbatim by an independent transcription company; transcripts were checked for accuracy by listening again to each recording. The NVivo 10 software package was used to manage the data.

Data collection and analysis ran concurrently throughout the study. Analysis of early transcripts informed the interview schedule for later interviews and each transcript was re-examined in light of subsequent interviews. A thematic analysis was conducted.[17] The first stage involved researchers familiarising themselves with the data through detailed reading of the transcripts followed by line-by-line coding.[18] Field notes taken during data collection were used throughout analysis to enhance the reflective process. Several quality control measures were employed to ensure trustworthiness of the data. A proportion of the transcripts (20%) were coded independently by three researchers, before coming together to compare their analysis. Data analysis and emerging themes were also discussed amongst the wider research team, which included individuals with clinical expertise in general practice and hepatology.

Findings

For peer review only - http://bmjopen.bmj.com/site/about/guidelines.xhtml

For peer review only

Twenty five GPs were interviewed. The majority had been qualified as GPs for five or more years, but few (4/25) had any specialist hepatology or gastroenterology training or experience. Participant characteristics are shown in table 1.

Table 1 here

Four themes were identified from the data analysis: the role of the GP, acknowledging and accepting end-of-life, collaborative care pathways, and social relationships and consequences. The quotations presented below are illustrative, representing typical participant responses and demonstrating the varied viewpoints.

The role of the GP

In this study, few of the interviewees had extensive first-hand experience of managing patients with liver disease at the end-of-life. Those who did, reported that they managed such cases infrequently, and some years may go by without them seeing a case.

> *"[We manage] a lot of dying people, but not from the hepatology point of view. I don't know if they tend to be managed in hospital predominantly more than in primary care? That's a possibility, I guess."* (GP 7)

Some of the interviewees attributed their lack of expertise and experience of caring for liver patients at the end-of-life to a reluctance amongst hospital clinicians to relinquish control.

For peer review only - http://bmjopen.bmj.com/site/about/guidelines.xhtml

"There are some conditions, like liver disease, renal failure, they [patients] all just end up dying in hospital for some reason. I don't know whether it's the hospital consultants that don't want to let them go home… They need to let go and make sure there's a palliative care plan in place…they don't do it." (GP 3)

The limited contact between GPs and patients dying with liver disease was attributed to an unpredictable disease trajectory with periods of stability and decompensation, and to patients remaining under the care of hospital services in their last weeks and months. The GPs in this study shared a view that end-of-life care is a core component of primary care, and interviewees questioned how appropriate it was for specialist hospital clinicians to take a lead in palliative care. Patients with liver disease were not regarded as distinct or different from patients dying with other conditions, and a number of GPs expressed a desire for greater involvement in their end-of-life care. Some participants implied that primary care involvement may support more patients to die at home, rather than in hospital.

"I think primary care probably is best placed, in most cases, to look after people- well not only for that [liver disease], for most end-of-life care issues. So, yeah, I think the GP is probably the most important person in the sense that they can bear in mind what the specialists have advised, but at the end of the day, try and keep some of these patients at home rather than having to have them admitted acutely." (GP 14)

For peer review only - http://bmjopen.bmj.com/site/about/guidelines.xhtml

Acknowledging and accepting end-of-life

Judging when a patient with liver disease is nearing the end-of-life was perceived to be a particular challenge. Communication about prognosis and the age of patients were identified as important factors. Some of the GPs reflected on how management decisions taken in hospital send out messages that influence care provided in the community. Continuing to pursue active treatment may convey optimism about the patient's life expectancy. Specifically, GPs referenced occasions where patients had been placed on the waiting list for a liver transplant, which the patient saw as offering them a second chance at life, even though they were critically unwell and may die whilst waiting for an organ. Patients with end stage liver disease are often younger than the typical palliative care patient.[19] It may be that clinicians are more reluctant to give up on active treatment for younger patients,[20] whilst patients and families may also struggle to accept that the end-of-life is approaching.[21]

> *"Those patients where it's a, kind of, grey area about whether they're end-of-life or not. And I think that mainly stems from the fact that if it's a young patient, it's more difficult for health care professionals, the patients themselves, and families, to actually accept that the person's dying"* (GP1)

Mixed or uncertain messages may mean that care is compromised, if no one engages the patient in discussions about the end-of-life, and a palliative approach is never considered.

For peer review only - http://bmjopen.bmj.com/site/about/guidelines.xhtml

> *"I suppose, looking back it really was palliative care but they [secondary care] put him on the transplant list because he's given up alcohol and there was still this hope. So therefore we didn't really realise he was going to die as quickly as he did"* (GP 11)

There was a shared feeling amongst interviewees, that specialists should provide clear messages about patients' prognoses, so that GPs can adopt an appropriate management plan. At present, hospital specialists were perceived as failing to take responsibility for identifying patients as end-of-life, and this had a detrimental impact on primary care.

> *"I feel that it should be made compulsory for the secondary care, tertiary care sectors, when they discharge, or when they're seen in the patient clinic, [to] prognosticate, … then we can initiate also, the discussion with the patient, in a much more positive way."* (GP 12)

Collaborative care pathways

Supporting patients with liver disease was seen as a collaborative effort, with GPs acknowledging their need for specialist guidance, particularly when managing end-of-life complications. A small number of respondents mentioned hepatic encephalopathy as a challenge in the management of end-of-life liver patients, and a potential source of distress for relatives. The interviewees suggested that they would benefit from further training to deal with this complication. Ascites was the most commonly mentioned symptom experienced by patients with end-stage liver

For peer review only - http://bmjopen.bmj.com/site/about/guidelines.xhtml

disease, requiring drainage in hospital. Experiences of GPs in this study suggest that ease of access to this procedure was highly variable. In some areas, pathways had been negotiated and patients could be directly admitted to an appropriate ward. In others, GPs described their concern at having to send patients to accident and emergency (A&E) departments. Failure to arrange prompt access to treatment caused distress and was a major source of adverse experiences during end-of-life care.

> *"We had a nightmare. He was building up litres of ascitic fluid on his tummy every week or week to 10 days, and every time the hospital had to send him acutely, new, to A&E and he had to sit in A&E for hours. I was speaking to the liver specialist ... He needed regular reviews and eventually they agreed to do it two weekly but even that wasn't enough, it was building up and he was ending up going in as an emergency every week."* (GP 11)

Where appropriate care pathways were not in place, interviewees suggested that they were needed, to reassure the patient and GPs that support is available when required.

A number of participants suggested that a specialist nurse may hold the key to more collaborative management of liver patients. They could act as an intermediary between primary and secondary care, negotiating priorities and ensuring effective and easy communication.

> *"It often helps when there is direct access to, say, a nurse specialist in a field, or there is some other point of contact in secondary care*

For peer review only - http://bmjopen.bmj.com/site/about/guidelines.xhtml

that say a family or the patient themselves can call directly for advice." (GP 14)

Although some GPs had encountered specialist nurses working in this type of role, this was not a common experience. Unfavourable comparisons were made between the services available for patients with liver disease, and other conditions, such as cancer. Participants highlighted the potential benefit to patients and families, of having a specialist point of contact in the community, including prompt access to advice and alleviation of fears and concerns.

Social relationships and consequences

GPs in this study argued that people with liver disease had many of the same primary care needs as patients with other life-limiting conditions. However, the severity of symptoms in end-of-life liver disease was felt to be different. Some of the GPs acknowledged the potentially damaging impact on the patient's family, of seeing their relative die at home.

"I think there is quite a strong push to keep people at home. Whether that's right or wrong, I don't know really. If they've got ascites or portal hypertension, you know, they've got the risk of vomiting blood and all the rest of it. Or they have been vomiting blood. I'm not massively keen on keeping people at home because it's just a rubbish picture in the mind of everybody, I think, you know, the family left behind." (GP 17)

For peer review only - http://bmjopen.bmj.com/site/about/guidelines.xhtml

Families were perceived to be in need of support themselves, which was an additional role for primary care. GPs described examples of relatives requiring frequent contact and reassurance as the patient's condition deteriorated. The GPs in this study differed in their attitudes towards these demands. Some took a holistic view to the management of palliative patients, believing that these were part of the standard practice of primary care.

> *"I think when we talk about palliative care it's not just a single person who's the patient, it's about supporting and managing the family and helping them through that bereavement stage because it starts right at the diagnosis and they have to go through that journey. Death is a part of life and giving them that support."* (GP 10)

However, others felt that attending to the needs of patients' families was an extra burden on their already overstretched resources.

Limited social support and unfavourable social circumstances were often mentioned as significant issues for patients with liver disease, particularly when alcohol or drug misuse were factors. Several GPs referred to the 'chaotic' lifestyles of this patient group, and resulting vulnerability to social isolation. Behaviours associated with addiction were perceived to lead to the breakdown of the patients' social networks, leaving few, if any people to provide support or care.

> *"the demographics of the alcohol dependent ones, who have often, for various reasons and due to the nature of their disease, have become quite isolated, they have not got many people around them*

For peer review only - http://bmjopen.bmj.com/site/about/guidelines.xhtml

For peer review only

and so they don't have that support. They require much more organisation and support in the background, so we make sure that they do have that support." (GP 10)

Without alternative sources of support, socially isolated patients were believed to place extra demands on GPs and other health services. Even when social networks were maintained, there could be a dearth of responsible caregivers, as friends and family often shared the problems of addiction and poor health.

"I can think of a couple of our households where maybe spouses and partners may have liver cirrhosis themselves. I can think of two couples – well, one person who died two years ago. His wife has chronic liver disease as well." (GP 4)

One of the most important consequences of social isolation, was that patients had fewer choices over where they spent the end of their life. Without anyone to monitor their condition, they were more likely to be admitted to hospital, and die there.

Liver disease is a potentially stigmatising condition, particularly when the underlying cause is alcohol or substance misuse. Several of the GPs suggested that there is often an assumption within the patient's community that liver disease is self-induced and they were culpable, even when substance misuse or alcohol are not factors.

For peer review only

For peer review only - http://bmjopen.bmj.com/site/about/guidelines.xhtml

"I think it's a huge problem for people that have liver disease and look like they have liver disease and people assume it's related to alcohol when, in fact, it might be due to auto-immune causes or other forms of cancer or something like that or hepatitis as well." (GP 23)

This assumed culpability has implications for the degree of support and sympathy that the patient, and their families, receive. GPs also suggested that stigma could hinder patients' acceptance of their prognosis, which in turn made the management of their condition more challenging. As such, care of liver patients should include psychological and social services.

"I think, inevitably and sadly, there is a stigma associated with liver disease, and hence, that's why the psychological support is really important" (GP 25)

However, some commented that stigmatisation occurred early in the patients' illnesses, and to address this, changes would be needed well before end-of-life care was being considered.

Discussion

This study provides insights into the challenge faced by general practitioners providing end-of-life care for patients with chronic liver disease. Many GPs expressed a desire to be more closely involved, but identified a number of factors that constrained their ability to contribute. These fell into three main areas; those relating directly to the condition, (symptom management and the need to combine a palliative care approach with ongoing medical interventions); issues arising from

For peer review only

patients' social circumstances (stigma, social isolation and the social consequences of liver disease); and deficiencies in the organisation and delivery of services. Collaborative working with support from specialist hospital clinicians was regarded as essential, with GPs acknowledging their lack of experience and expertise in this area.

A majority of interviewees had little direct experience of patients dying of liver disease and as a consequence, they may not have been familiar with all the management challenges of end stage liver disease. For example, hepatic encephalopathy is a common concern in the care of end stage liver patients, yet few of the interviewees discussed it. This is not surprising, as primary care clinicians would seldom have responsibility for managing hepatic encephalopathy, if they are not dealing day to day with end-of-life care for liver disease patients.

Strengths and limitations

To our knowledge this is the first study to focus on the experiences of primary care physicians in managing patients with end-stage liver disease. Our interviewees were drawn from rural and urban areas in five different regions in England, and working with a diverse range of communities. The relatively large number of GP participants and varying levels of experience, expertise and interest in the subject, is a particular strength of the study. With our qualitative design, we were not seeking generalisability, but the diversity of the participants increases our confidence that we have not overlooked important issues.

For peer review only - http://bmjopen.bmj.com/site/about/guidelines.xhtml

For peer review only

The majority of interviews were conducted by telephone, which may explain the ease and speed with which we recruited participants, despite not offering any financial incentives. Use of the telephone is thought to have promoted unguarded responses, but we acknowledge that it can be more difficult to develop rapport in the absence of nonverbal cues and other facets of face to face communication. However, we do not believe that this was a problem, as GPs provided rich and insightful accounts of their experiences.

Comparison with other work

Our findings are consistent with recent research from Scotland that included interviews with eight GPs along with other health care professionals. Communication with secondary care, lack of expertise and limited confidence in prognostication were all identified as concerns.[15] Accurate assessment of prognosis in liver disease is difficult given the unpredictable disease course. In some aspects this is similar to other diseases characterised by episodes of decompensation, such as heart failure. However, liver disease presents the additional challenge that recompensation and improved liver function may be achieved in certain patients, such as those who achieve abstinence from alcohol. A recent review of palliative care guidelines in heart failure and chronic obstructive pulmonary disease described wide variation in how patients are identified for palliative care, and attributed this, in part, to the unpredictable disease course and the consequences for care planning.[22] In common with liver disease, acknowledgement and development of end-of-life care has been relatively recent for these conditions.[22]

For peer review only - http://bmjopen.bmj.com/site/about/guidelines.xhtml

For peer review only

The GPs in our study agreed that, at the end-of-life, patients with liver disease ideally need primary care and hospital specialists to work closely together. GPs are more likely to have an established relationship with the patient and a greater understanding of their social situation and needs, whereas specialists offer expert knowledge on liver disease and treatment options. They highlighted the importance that primary care physicians place on being able to provide a coordinating role, but only when supported by members of the specialist teams. Managing complex and unusual symptoms, or judging when to introduce a palliative care approach, for example, all benefit from collaboration. The advantages of a multidisciplinary approach have already been well documented in the palliative care literature.[23 24] Several recent reviews on end-stage liver disease have also advocated this approach.[4 5 14]

This study highlights the complexity of caring for patients with end-stage liver disease. Expertise in acute medicine and palliative care are essential, but patients and families also need sensitive and practical responses to their psycho-emotional and social concerns, including stigma related to the perceived self-inflictedness of the disease, social isolation and lack of income. Such generalist expertise and a holistic, person-centred approach are the foundations of primary care. Community-based services already play an important role at the end-of-life for patients with many different, complex conditions. However, this seldom includes people dying with liver disease. Greater involvement of community services would be expected to enhance the quality and appropriateness of palliative and terminal care for these patients. As the number of deaths from chronic liver disease increases, it may be increasingly necessary, in order to limit the burden on hospital teams. Innovations,

For peer review only - http://bmjopen.bmj.com/site/about/guidelines.xhtml

such as the development of clear patient pathways, specialist heptology nurses in the community or district nurses trained to deal with liver disease complications, all require resources. Specialist treatments such as paracentesis could be delivered in locations such as community hospitals or hospices, where they are available, to reduce disruption to patients' lives.

(Box 1 here)

In recent years, UK health policy has increasingly promoted patient choice; an ability to deliver end of life care in the patient's preferred location and facilitate choice in place of death are used as markers of care quality, with death at home often an implicit goal of palliative care.[25 26] GPs in this study expressed some scepticism that home death is always the best option for patients with liver disease, or their families. Concerns centred around the nature of the symptoms and clinical input needed to manage them, which were potentially distressing for families to observe. Balancing the wishes of patients, families and clinical carers is a fundamental part of end of life care. More in depth enquiry to elicit patient, family and professional views and experiences of place of death in liver disease would help to clarify the resources required to ensure death at home is acceptable and achievable.

Conclusion

Our study suggests that end-of-life care for patients with liver disease requires attention. Liver disease appears to pose management challenges in end-of-life care with a combination of complicated social situations and symptoms. Services tailored for these patients should build on the similarities with other conditions, but also reflect the differences. The adverse social consequences of illness for these patients

For peer review only - http://bmjopen.bmj.com/site/about/guidelines.xhtml

and their families, may be particularly significant. Further research is needed to fully understand the burden on families and services. As health services seek greater integration with social care, improving care for patients with end-stage liver disease should be a priority.

For peer review only

Acknowledgements

We are grateful to the participants who generously gave their time to this study.

Ethics Approval

This study involved no patients and was assessed by the Health Research Authority and Newcastle University Research Ethics (Ref 188275).

Authors contribution

BH, EK, MH, JO, and CE designed the study. HS carried out the interviews. HS undertook the main analysis supported by BH and HJ. HS, HJ and BH drafted the manuscript, and all authors commented and approved the final version.

Funding statement

This work was supported by the NIHR School for Primary Care grant number 262.

Competing interests

We have read and understood BMJ policy on declaration of interests and declare that we have no competing interests.

Data sharing agreement

We do not have any additional unpublished data that can be shared.

References

1. World Health Organisation. WHO European health for all database. 2012. http://data.euro.who.int/ (accessed 22 January 2017).
2. Office for National Statistics. Deaths registered in England and Wales: 2015. 2016; (9th September 2016).

https://www.ons.gov.uk/peoplepopulationandcommunity/birthsdeathsandmarriages/deaths/bulletins/deathsregistrationsummarytables/2015 (accessed 22 January 2017).
3. University of Washington. The Liver Imaging Atlas: An Online Reference for Liver Imaging. 2017; 2017(22 January 2017). liveratlas.org (accessed 22 January 2017).
4. Boyd K, Kimbell B, Murray S, et al. Living and dying well with end-stage liver disease: Time for palliative care? *Hepatology* 2012;55(6):1650-51. doi: 10.1002/hep.25621
5. Roth K, Lynn J, Zhong Z, et al. Dying with end stage liver disease with cirrhosis: insights from SUPPORT. Study to Understand Prognoses and Preferences for Outcomes and Risks of Treatment. *Journal of the American Geriatrics Society* 2000;48(5 Suppl):S122-30.
6. Kimbell B, Murray SA. What is the patient experience in advanced liver disease? A scoping review of the literature. *BMJ Supportive & Palliative Care* 2013;bmjspcare-2012 doi: 10.1136/bmjspcare-2012-000435
7. National End of Life Care Intelligence Network. Deaths from Liver Disease: Implications for end of life care in England. 2012. http://www.endoflifecare-intelligence.org.uk/resources/publications/deaths_from_liver_disease (accessed 27 Feburary 2017).
8. Kendrick E. Getting it Right Improving End of Life Care for People Living with Liver Disease. London: National End of Life Care Programme, 2013.
9. European Association for the Study of the Liver. EASL clinicial practice guidelines: liver transplantation. *Journal of Hepatology* 2016;64(2):433-85.
10. Poonja Z, Brisebois A, van Zanten SV, et al. Patients with cirrhosis and denied liver transplants rarely receive adequate palliative care or appropriate management. *Clinical Gastroenterology and Hepatology: The Official Clinical Practice Journal of the American Gastroenterological Association* 2014;12(4):692-98. doi: 10.1016/j.cgh.2013.08.027
11. Potosek J, Curry M, Buss M, et al. Integration of Palliative Care in End-Stage Liver Disease and Liver Transplantation. *Journal of Palliative Medicine* 2014;17(11):1271-77.
12. Gott M IC, Gardiner C, Richards N, Cobb M, Ryan A, Noble B, Bennett M, Seymour J, Ward S, Parker C. Transitions to palliative care for older people in acute hospitals: a mixed-methods study. . *Health Services and Delivery Research* 2013;1(11)
13. Larson AM. Palliative Care for Patients with End-Stage Liver Disease. *Current Gastroenterology Reports* 2015;17(5):18. doi: 10.1007/s11894-015-0440-6
14. Walling AM, Wenger NS. Palliative care and end-stage liver disease. *Clinical Gastroenterology and Hepatology: The Official Clinical Practice Journal of the American Gastroenterological Association* 2014;12(4):699-700. doi: 10.1016/j.cgh.2013.11.010
15. Kimbell B, Boyd K, Kendall M, et al. Managing uncertainty in advanced liver disease: a qualitative, multiperspective, serial interview study. *BMJ open* 2015;5(11):e009241. doi: 10.1136/bmjopen-2015-009241
16. Office for National Statistics. National Survey of Bereaved People (VOICES). 2015. (accessed 27 Feburary 2016).
17. Braun V, Clarke, V. Using thematic analysis in psychology. *Qualitative Research in Psychology* 2006;3(2):77-101. doi: 10.1191/1478088706qp063oa
18. Ritche J, Lewis J. Qualitative Research Practie: A Guide for Social Science Students and Researchers. London: SAGE Publications 2003.
19. Office for National Statistics. Deaths registered in England and Wales (Series DR): 2015. 2016. (accessed 31 May 2017).
20. Department of Health. The impact of patient age on clinical decision-making in oncology. 2012.
21. Thompson GN, Chochinov, H.M., Wilson, K. G., McPherson, C. J., Chary, S., O'Shea, F. M., Kuhl, D. R., Fainsinger, R. L., Gagnon, P.R., Macmillan, K. A. Prognostic Acceptance and the Well-Being of Patients Receiving Palliative Care for Cancer. *Journal of Clinical Oncology* 2009;27(34):5757-62.

For peer review only - http://bmjopen.bmj.com/site/about/guidelines.xhtml

22. Siouta N, Van Beek K, van der Eerden ME, et al. Integrated palliative care in Europe: a qualitative systematic literature review of empirically-tested models in cancer and chronic disease. *BMC Palliative Care* 2016;15:56. doi: 10.1186/s12904-016-0130-7
23. Davidson PM, Paull G, Introna K, et al. Integrated, collaborative palliative care in heart failure: the St. George Heart Failure Service experience 1999-2002. *The Journal of Cardiovascular Nursing* 2004;19(1):68-75.
24. Yohannes AM. Palliative care provision for patients with chronic obstructive pulmonary disease. *Health and Quality of Life Outcomes* 2007;5:17. doi: 10.1186/1477-7525-5-17
25. Department of Health. End of Life Care Strategy. 2008. https://www.gov.uk/government/publications/end-of-life-care-strategy-promoting-high-quality-care-for-adults-at-the-end-of-their-life (accessed 13th June 2017).
26. National Institute for Health and Care Excellence. NICE Quality Standards on End of Life Care. 2011. https://www.nice.org.uk/guidance/qs13 (accessed 13th June 2017).

For peer review only

Table 1: Participant characteristics

Characteristic	Number of GPs
**Sex**	
Male	12
Female	13
**Years of experience as GP**	
<5 years	5
5-10 years	10
16-25 years	9
>25 years	1
**Specialist hepatology/gastroenterology experience or training**	
Yes	4
No	21
**Size of practice**	
<5,000	5
5-10,000	9
10,000-15,000	9
>15,000	2
**Geographical area**	
North West London	7
Wessex	8
North East and North Cumbria	5
Yorkshire and Humber	1
Thames Valley and South Midlands	4

Box 1

For peer review only - http://bmjopen.bmj.com/site/about/guidelines.xhtml

Next steps in primary end of life care for liver disease: GP perceptions of areas for development

- Education and training in symptom management for end stage liver diease
- Collaborative care pathways between primary care and hepatologists
- Service delivery that takes into account patient and family social circumstances and stigma
- Provision of support for family caregivers
- Consideration / research into the role of home death

For peer review only

For peer review only - http://bmjopen.bmj.com/site/about/guidelines.xhtml

No	Item	Guide questions/description	
**Domain 1: Research team and reflexivity**			
Personal Characteristics			
1.	Interviewer/facilitator	Which author/s conducted the interview or focus group?	HS
2.	Credentials	What were the researcher's credentials? *E.g. PhD, MD*	PhD, MSc, Bsc
3.	Occupation	What was their occupation at the time of the study?	Research Associate in the Institute of Health and Society, Newcastle University
4.	Gender	Was the researcher male or female?	Female
5.	Experience and training	What experience or training did the researcher have?	Conducting interviews and analysing data from patients, clinicians and

No	Item	Guide questions/description	
			family members
Relationship with participants			
6.	Relationship established	Was a relationship established prior to study commencement?	In some areas authors knew the representatives from the clinical research networks who assisted with recruitment. In other areas there was no relationship. There were no prior relationships with GP participants.
7.	Participant knowledge of the	What did the participants know about the researcher?	Reasons for

No	Item	Guide questions/description	
	interviewer	e.g. *personal goals, reasons for doing the research*	doing the research were provided in information sheets sent to participants before the interviews.
8.	Interviewer characteristics	What characteristics were reported about the interviewer/facilitator? e.g. *Bias, assumptions, reasons and interests in the research topic*	HS is a medical Sociologist with a specialist interest in end of life issues
**Domain 2: study design**			
Theoretical framework			
9.	Methodological orientation and Theory	What methodological orientation was stated to underpin the study? *e.g. grounded theory, discourse analysis, ethnography, phenomenology, content analysis*	Thematic analysis See page 6 of the article

For peer review only - http://bmjopen.bmj.com/site/about/guidelines.xhtml

No	Item	Guide questions/description	
Participant selection			
10.	Sampling	How were participants selected? *e.g. purposive, convenience, consecutive, snowball*	Purposive sampling<br>See page 5 of the article
11.	Method of approach	How were participants approached? e.g. *face-to-face, telephone, mail, email*	Face-to-face and telephone<br>See page 5 of the article
12.	Sample size	How many participants were in the study?	25<br>See page 5 of the article
13.	Non-participation	How many people refused to participate or dropped out? Reasons?	8 GPs expressed interest in participating in the study but later dropped out citing lack of time to participate.

No	Item	Guide questions/description	
Setting			
14.	Setting of data collection	Where was the data collected? e.*g. home, clinic, workplace*	Over the telephone, workplace
15.	Presence of non-participants	Was anyone else present besides the participants and researchers?	No one was present expect the participants and researchers
16.	Description of sample	What are the important characteristics of the sample? *e.g. demographic data, date*	Wide range of clinical experience from GP trainee to >30 years experience Participant characteristics are presented in Table 1
Data collection			

No	Item	Guide questions/description	
17.	Interview guide	Were questions, prompts, guides provided by the authors? Was it pilot tested?	Yes, an interview schedule was developed. This was pilot tested with one interviewee See page 5 of the article
18.	Repeat interviews	Were repeat interviews carried out? If yes, how many?	No repeat interviews were conducted
19.	Audio/visual recording	Did the research use audio or visual recording to collect the data?	Audio recording see page 6 of the article
20.	Field notes	Were field notes made during and/or after the interview or focus group?	Yes see page 6 of the article

No	Item	Guide questions/description	
21.	Duration	What was the duration of the interviews or focus group?	15-50 minutes
22.	Data saturation	Was data saturation discussed?	Yes, data collection was stopped when new themes were no longer emerging from the data. See page 5 of the article
23.	Transcripts returned	Were transcripts returned to participants for comment and/or correction?	No
**Domain 3: analysis and findings**z			
Data analysis			
24.	Number of data coders	How many data coders coded the data?	3 See page 6 of the article

No	Item	Guide questions/description	
25.	Description of the coding tree	Did authors provide a description of the coding tree?	No, documentation detailing development of themes is available upon request.
26.	Derivation of themes	Were themes identified in advance or derived from the data?	Derived from the data See page 6 of the article
27.	Software	What software, if applicable, was used to manage the data?	NVivo10 See page 6 of the article
28.	Participant checking	Did participants provide feedback on the findings?	No
Reporting			
29.	Quotations presented	Were participant quotations presented to illustrate the themes / findings? Was each quotation identified? e.g. *participant number*	Yes See pages 7-15 of the article

No	Item	Guide questions/description	
30.	Data and findings consistent	Was there consistency between the data presented and the findings?	Yes there is consistency between the data presented and the findings. See pages 7-15 of the article
31.	Clarity of major themes	Were major themes clearly presented in the findings?	Yes, major themes are presented clearly in the article see pages 7-15.
32.	Clarity of minor themes	Is there a description of diverse cases or discussion of minor themes?	Yes, there are descriptions of diverse and minor themes in the article, see pages 7-15.
